# Effects of Feeding Sources and Different Temperature Changes on the Gut Microbiome Structure of *Chrysomya megacephala* (Diptera: *Calliphoridae*)

**DOI:** 10.3390/insects16030283

**Published:** 2025-03-08

**Authors:** Fernand Jocelin Ngando, Haojie Tang, Xianqi Zhang, Xiangyan Zhang, Fengqin Yang, Yanjie Shang, Jifeng Cai, Yadong Guo, Lei Zhao, Changquan Zhang

**Affiliations:** 1 Department of Forensic Science, School of Basic Medical Sciences, Central South University, Changsha 410013, China212144@csu.edu.cn (Y.G.); 2 Institute of Forensic Science of China, Beijing 100038, China

**Keywords:** *calliphoridae*, feeding sources, gut microbiome, lifespan, constant and variable temperatures

## Abstract

*Chrysomya megacephala* (Diptera: *Calliphoridae*), commonly referred to as the oriental latrine fly, is a synanthropic blowfly species frequently associated with decomposing organic matter. This study sought to investigate the influence of various feeding substrates and temperature conditions, specifically constant temperatures of 15, 25, 35 °C, and variable temperatures averaging 23.31 °C, on the gut microbiome of *C. megacephala*. The microbiome analysis was conducted using the Illumina HiSeq platform for 16S rRNA gene sequencing in Changsha, China. Across all experimental conditions, the gut microbiome of *C. megacephala* yielded 1257 operational taxonomic units (OTUs), which were categorized into 26 phyla, 72 classes, 165 orders, 270 families, 516 genera, and 794 species. The study showed significant differences in the gut microbiome of *C. megacephala* between different feeding sources and temperature conditions across the lifespan. Low temperature had the potential to reduce the proportion abundance of bacterial communities in the gut microbiome, while high and variable temperature increased them. Metabolism was the main predicted function with diverse phenotypic characters in the gut microbiota of *C. megacephala*. The presence of diverse bacterial phenotypes in the gut microbiome of *C. megacephala* highlights its significant interest for medicine and offers promising applications in industry and agriculture.

## Simple Summary

*Chrysomya megacephala* (Diptera: *Calliphoridae*), a synanthropic blowfly species, is frequently found on decaying organic matter, including human or animal remains, feces, and garbage dumps. In this study, we examined the impact of different feeding sources and temperature variations, including constant temperatures of 15, 25, and 35 °C, and the variable temperatures with an average of 23.31 °C, on the gut microbiome of *C. megacephala*. Interestingly, the richness and diversity of the gut microbiome in *C. megacephala* were considerably influenced by feeding sources and temperature variations. The presence of diverse bacterial phenotypes in the gut microbiome of *C. megacephala* highlights its significant interest for medicine and offers promising applications in industry and agriculture.

## 1. Introduction

*Chrysomya megacephala* (Fabricius, 1794) (Diptera: *Calliphoridae*) or the oriental latrine fly [1], is a widely distributed synanthropic blowfly species that thrives on decomposing organic matters [2,3,4]. Its significance extends to various fields such as forensics, medicine, veterinary science [5,6,7,8,9,10,11], the food industry, and organic waste recycling [2]. Same as most flies, *C. megacephala* may be a potential microorganism vector, because it is ubiquitous and synanthropic [12]. Symbiotic interactions between insects and microorganisms contribute to vital biological functions [13,14,15,16,17,18,19,20]. A substantial body of research has explored the intricate relationships between these organisms [12,15,21,22,23,24,25,26,27,28,29]. Previous research demonstrated that the gut microbiome of *C. megacephala* is highly diverse, which varies across life stage. And in the pupal stage, *Wolbachia* and *Ignatzschineria* coexist at the genus level, which is a rarely known feature [30,31].

The gut microbiome is a complex assemblage of bacteria which can shape the biological structure of the host organism [13]. It can consist of a diverse community of microorganisms or just a single dominant bacterial species [32]. These bacterial communities are subjected to the host’s development and physiology, establishing a mutually beneficial relationship [26,33]. Previous studies have revealed that insects’ bacterial community structure can be affected by a variety of endogenous and exogenous factors [34], and the dominant factors are feeding sources [35,36,37,38], which can influence the structure and community composition of the gut microbiome in insects [39,40,41]. Different feeding sources have varying macronutrient compositions, resulting in the proliferation of specific microbial species in the host [41]. For instance, the bacterial community in the red palm weevil (*Rhynchophorus ferrugineus*) was found to be significantly influenced by feeding sources [42]. Similarly, diets have significantly influenced the gut microbiome community in the housefly larvae [43], and *Blattella germanica* [44]. *Bactrocera tryoni* (Diptera: *Tephritidae*) larvae fed on different diets revealed that *Enterobacteriaceae* dominated laboratory-reared tenerals from a colony fed a carrot-based larval diet, while *Acetobacteraceae* dominated mass-reared tenerals from a production facility colony fed a lucerne chaff based larval diet [45]. *Zeugodacus cucurbitae* (Coquillett) reared on the bran-based artificial diet displayed significant changes in the bacterial symbionts upon irradiation, drastically reducing the number of *Citrobacter*, *Raoultella*, and *Enterobacteriaceae*. At the same time, an increase was observed for members of *Enterobacter*, *Providencia*, and *Morganella* [46]. In contrast, Li et al. [41] found a relatively stable gut bacterial community in *Calliphora grahami* (Diptera: *Calliphoridae*), regardless of feeding sources.

Temperature is also an important environmental factor that plays a crucial role in shaping the life history and physiology of ectotherms like insects [47,48,49,50,51,52]. While research on the impacts of temperature variations on the gut microbiome of vertebrate ectotherms is extensively documented [53,54,55,56,57,58], however, it remains limited to invertebrate poikilotherms. Nevertheless, according to the few documented studies, elevated body temperature has been found to aid in insect infection resistance [59,60,61]. For example, in bumblebees infected with the trypanosomatid parasite *Crithidia bombi*, it was found that temperature changes influenced host and parasite performance, as well as the gut bacteria dynamic, all of which contributed to infection resistance [61]. During the diapause stage of *Nasonia vitripennis* (Walker), changes in environmental temperature, host nutrient levels, and downregulation of host immune genes were found to modify the gut microbiome [62].

Despite its medical, veterinary, and forensic significance, the gut microbiome of *C. megacephala* remains inadequately studied. Furthermore, the impact of temperature on its gut microbiome remains unclear. Given the projected rise in extreme environmental temperatures [63], it is crucial to comprehend the effects of temperature on the gut microbiome of *C. megacephala*.

Holometabolous insects undergo several physiological changes during the metamorphosis process. These changes involve tissue breakdown and reconstruction at different developmental stages [64,65,66]. Furthermore, the larval, pupal, and adult stages of insects experience distinct feeding activities, resulting in varied gut environments that can affect their gut bacterial symbionts [13,41]. Therefore, in order to analyze the stability of the gut bacterial colony in *C. megacephala*, three variables were selected: temperatures, feeding sources, and life history traits.

In this study, we examined the gut microbiome of *C. megacephala* at three constant (15, 25, and 35 °C) and variable (averaging 23.31 °C) temperatures. By analyzing the hypervariable V3 and V4 regions of the bacterial gene 16S rRNA amplicons, we compared the gut bacterial composition, diversity, and abundance across different developmental stages, from larvae to adults. Furthermore, the study also investigated the effects of temperatures and feeding sources on the gut bacteria. The larvae in each treatment were fed daily with fresh pork lungs or with artificial diet cubes which consisted of a protein base made with fish meal and wheat bran at a constant temperature of 25 °C with 70% RH and a 12:12 h L/D photoperiod cycle. These findings shed light on the impact of temperatures and feeding sources on the gut microbiome of *C. megacephala*.

## 2. Materials and Methods

### 2.1. Fly Colony and Rearing Process

In September 2021, adults of *C. megacephala* were collected from pig carcasses using nylon nets as fly traps in Xi Hu Park in Changsha, Hunan Province, China City (28°12′ N, 112°58′ E). They were anesthetized at −20 °C for 1–2 min to facilitate species identification through morphological analysis using Zeiss AxioCam 208 Color Microscopy (Carl Zeiss Microscopy GmbH, Jena, Germany), guided by a forensic entomologist [67]. Additionally, a 658 bp fragment of the long cytochrome oxidase I (COI) gene was amplified and sequenced using forward (COI-J-1460: 5′-TACAATTTATCGCCTAAACTTCAGCC-3′) and reverse (COI-N-2800: 5′-CATTTCAAGCTGTGTAAGCATC-3′) primers to confirm the species identification [68,69]. The obtained sequences were deposited to GenBank under accession numbers GenBank No. OR739421 and OR739422. Identified specimens were bred using established techniques in a nylon box (35 × 35 × 35 cm) and transferred to an artificial climate cage (250A GPL, Shen Zhen Ren Gong Ltd., Tianjin, China) [70]. The flies were maintained at a temperature of 25 °C, with a 70% relative humidity, and a photoperiod cycle of 12:12 h light/dark. They were provided with water and milk powder as nourishment and bred for fifth generations with a population size of 2000–3000 specimens before the study.

Three artificial climate incubators (LRH-250-GSL, Taihong Co., Ltd., Shaoguan, China) were set at constant temperatures of 15 °C, 25 °C, and 35 °C with a relative humidity (RH) of 70% and a 12:12 h light-dark (L/D) photoperiod cycle. The variable temperature conditions were based on local meteorological data from Hunan Province, southeast China. A GPS temperature humidity data logger (GPS-6, Elitech, Co., Ltd., Suzhou, China) recorded the ambient temperature during May 2022, ranging from 21.0 °C to 25.4 °C, with an average of 23.31 °C. The relative humidity ranged from 46.8% to 75.2%. In addition, two supplementary temperature boxes were set up: one for pig lung feeding source and another for wheat bran plus fish meal feeding source. Both were maintained at 25 °C with a RH of 70% and a 12:12 h L/D photoperiod cycle.

### 2.2. Sample Collection

Approximately 50 g of fresh pork lungs and 100 g of wheat bran plus fish meal in Petri dishes were introduced to the fly breeding box to induce oviposition. After 2 h, we collected 2000–3000 eggs laid by gravid *C. megacephala* females. Each mass 4500–600 eggs were raised in a bowl with fresh pork lungs and artificial diet, placed in sand-filled containers, and transferred to fly-rearing cages until pupation.

After five generations, we collected approximately 100 eggs of *C. megacephala* specimen from each feeding source, which consisted of pig lung and wheat bran supplemented with fish meal. We then collected 30 larvae specimens from the first and second instar stages from each feeding source. Additionally, at the third instar stage, we collected 30 specimens from the early, late, and wandering stages. Furthermore, we sampled 30 pupae at the prepupal, early, and late stages from different feeding sources. Finally, at the adult stage, we collected 30 specimens at the early and late stages from varying feeding sources.

Under different temperature conditions, around 50% of larvae reached the third instar, 60 larvae were collected from each group and labeled as “larvae early”. This time was set as zero days (0D). Later, when all larvae reached maturity, another 60 were collected as “larvae late”. At the 50% wandering stage, 60 individuals were taken as wandering samples. Once larvae left food and began to pupate, 60 individuals were sampled as prepupae. Early pupal samples were collected at approximately 50% pupal emergence. When five pupae eclosed, 60 pupae were collected as “pupae late”. Before being fed, 60 adults were sampled as “adults early”. Adult late samples were taken 10–15 days later. All samples were stored at −20 °C in 15 mL centrifuge tubes. Each experiment was replicated three times. In total, 200 eggs, 120 larvae of first and second instar, 840 larvae, 420 wandering samples, 420 prepupal samples, 780 pupae, and 780 adults were collected across all treatment groups.

### 2.3. Sample Dissection and Gut Preparation

After removal from −20 °C refrigerator, samples were thawed and washed in tap water for 1–2 min, and then rinsed for 1 min in 70% alcohol. Any excess alcohol was removed with purified water for 2 min. Surface sterilization was performed by using a 0.05% sodium hypochlorite solution (NaClO) for 1 min. The eggs, as well as the first and second instar larvae samples, were directly suspended in 15 mL of 1× phosphate-buffered saline (PBS). On the other hand, the third instar larvae to adult late stages were dissected using tweezers (R’DEER, TST-11, ROBUST DEER TOOLS CO., Ltd., Guangzhou, China) and a Zeiss AxioCam 208 Color Microscope (Carl Zeiss Microscopy GmbH, Jena, Germany). The entire gastrointestinal tract (including the foregut, midgut, and hindgut) was then extracted and stored in a 1.5 mL Eppendorf tube with 1× PBS. All the samples were stored at −80 °C until DNA extraction.

### 2.4. Genomic DNA Preparation and 16S rRNA Sequencing

#### 2.4.1. DNA Extraction and PCR Amplification

Genomic DNA from the gut of eggs, larvae, pupae, and adult samples was extracted using the EZNA soil DNA KF kit (Omega Bio-Tek, Norcross, GA, USA) following the manufacturer’s protocol. The hypervariable regions V3 and V4 of the bacterial 16S rRNA gene were amplified with primer pairs 338F (5′-ACTCCTACGGGAGGCAGCAG-3′) and 806R (5′-GGACTACHVGGGTWTCTAAT-3′) on an ABI GeneAmp^®^ 9700 PCR Thermocycler (ABI, Los Angeles, CA, USA) [71]. The PCR reactions mix for each sample included 4 μL of 5× FastPfu Buffer, 2 μL of 2.5 mM dNTPs, 0.8 μL of each primer, 0.4 μL of FastPfu polymerase, 0.2 μL of BSA, 10 ng of template DNA, and ddH_2_O to a final volume of 20 μL. The PCR conditions were as follows: initial denaturation at 95 °C for 3 min, followed by 29 cycles of denaturation at 95 °C for 30 s, annealing at 53 °C for 30 s, extension at 72 °C for 45 s, and final extension at 72 °C for 10 min, with a final hold at 10 °C. Three replicates were amplified for each sample. The amplification products were visualized using 2% agarose gel electrophoresis, and the fragment lengths were approximately 750 bp. The PCR products were purified using the AxyPrep DNA Gel Extraction Kit (Axygen Biosciences, Union City, CA, USA), eluted with Tris-HCl buffer, and detected using a 2% agarose gel electrophoresis as per the manufacturer’s instructions. Quantification was performed using the QuantiFluor™-ST Blue Fluorescence Quantification System (Promega, Madison, WI, USA).

#### 2.4.2. Illumina MiSeq Sequencing and Data Processing

The amplicons were purified, grouped in equimolar marks, and subjected to paired-end sequencing on an Illumina MiSeq PE300 platform (Illumina, San Diego, CA, USA). Standard protocols provided by Majorbio Bio-Pharm Technology Co., Ltd. (Shanghai, China) were followed. Raw FASTQ files were then processed by dereplicating using a custom Perl script, quality-filtering with Fastp (version 0.19.6) [72], and merging with FLASH (version 1.2.11) [73]. Quality filtering removed low-scoring reads and those 50 bp or containing N-bases. Only overlapping sequences longer than 10 bp and with a maximum mismatch ratio of 0.2 were included in the analysis. Samples were distinguished using barcodes and primers, and the sequence direction was adjusted accordingly. Operational taxonomic units (OTUs) were clustered using UPARSE 7.0 [74,75] with a 97% sequence similarity level, and chloroplast or mitochondrial contamination was removed. Each OTU was taxonomically classified using the SILVA 16S rRNA gene database (version 138) and RDP Classifier (version 2.11) at a confidence threshold of 0.7 [76]. The metagenomic function of microbial communities was predicted using PICRUSt (Phylogenetic Investigation of Communities by Reconstruction of Unobserved States) (version 2.2.0). [77] And the Tax4Fun function prediction was used to infer the Kyoto Encyclopedia of Genes and Genomes (KEGG) pathway analysis of the OTUs. The BugBase tool (Version 0.1.0) was utilized to assess the phenotype prediction of the gut microbiome of *C. megacephala*.

Bioinformatic analysis was performed using the Majorbio Cloud platform (https://cloud.majorbio.com (accessed on 2 January 2025)). Mothur (version 1.30.2) was employed to generate rarefaction curves and alpha diversity indexes based on the OTU information [78]. The similarity among the microbial communities in different samples was evaluated using Principal Component Analysis (PCA) with Euclidean distances in R software (version 3.3.1). The statistical significance and percentage of difference explained by treatment were assessed using the PERMANOVA test in R software (version 3.3.1) and Qiime 1.9.1. To identify significantly abundant taxa from phylum to genus level, linear discriminant analysis effect size (LEfSe) was performed with an LDA score > 2 and a significant threshold of *p* < 0.05 [79,80]. The variance inflated factor (VIF) analysis, utilizing the VIF function from the R package, was used to address multicollinearity issues among the samples. The relationship between species and clinical factors was analyzed through distance-based redundancy analysis (db-RDA) using R software v3.3.1.

Statistical analysis was performed using Graph Pad Prism 9.4.1 and IBM SPSS Statistics 26. Wilcoxon rank-sum analysis was employed to evaluate the differences in the proportion of bacterial taxa across different temperatures, and the differences in the proportion of bacterial taxa between different feeding sources.

## 3. Results

### 3.1. Sample Size and Overview of 16S rRNA Gene Sequencing of the Gut Microbiota in C. megacephala

A total of 138 samples were generated to investigate the gut microbial communities in *C. megacephala* under different feeding sources and temperature conditions throughout its life cycle. Each sample represented a specific developmental stage and contained 10 specimens. CM-F represent the group fed with wheat bran plus fish meal, while CM-P represent the group fed with pork lung. The temperature treatments included 15 °C, 25 °C, 35 °C, and a variable temperature (averaging 23.31 °C). A total of 137 specimens were successfully sequenced after quality control (Tables S1 and S2).

**Figure 1:**
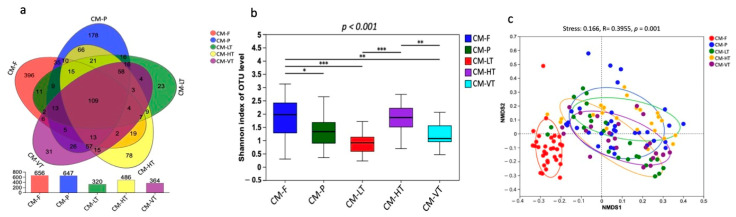
The gut bacterial composition of *C. megacephala* exhibited substantial variations under different experimental conditions. (**a**) Venn diagram showed the OTUs shared by CM-F, CM-P, LT, HT, and VT groups; (**b**) The Shannon diversity index showed the substantial variations in the gut bacterial composition of *C. megacephala* under different experimental conditions (* *p* < 0.05, ** *p* < 0.01, *** *p* < 0.001); (**c**) The non-metric multidimensional scaling (NMDS) with binary Jaccard distance showed the significant differences in the gut bacterial composition of *C. megacephala* between different experimental conditions (stress: 0.166, R = 0.3955, *p* = 0.001). Abbreviations: OTU: Operational Taxonomic Unit; CM-F: *C. megacephala* fed with wheat bran plus fish; CM-P: *C. megacephala* fed with pig lungs; CM-LT: *C. megacephala* maintained at low temperature; CM-HT: *C. megacephala* maintained at high temperature; CM-VT: *C. megacephala* maintained at variable temperature.

A total of 6,983,375 raw clean bacterial 16S rRNA gene sequences were obtained from Illumina HiSeq paired-end sequencing, with 3,986,015 valid sequences after quality control. The raw data generated were deposited in the NCBI Sequence Read Archive under accession: PRJNA997617. The samples from experimental conditions produced a total of 2,950,411,623 bp, with an average read length of 423 bp (Tables S1 and S2). The analysis of rank abundance showed by Shannon curves (Figure S1) demonstrated that all curves plateaued, indicating sufficient sampling. Using a 97% species similarity threshold, a total of 1257 OTUs ranging from 320 to 656 were identified from the 137 samples (Figure 1b). 109 OTUs were commonly shared by all experimental conditions, while 396, 178, 23, 78, and 31 OTUs were uniquely shared by CM-F, CM-P, LT, MT, HT, and VT groups, respectively (Figure 1a). The identified OTUs belonged to 1 domain, 1 kingdom, 26 phyla, 72 classes, 165 orders, 270 families, 516 genera, and 794 species.

**Figure 2:**
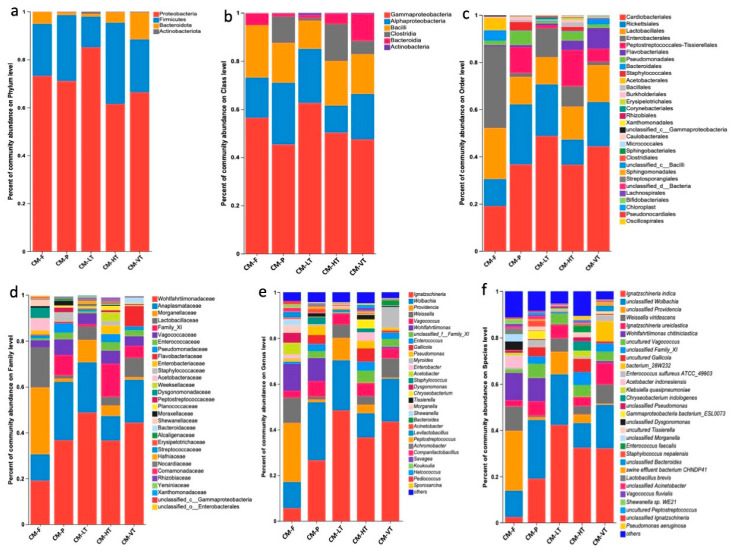
The dominant gut microbiota under all experimental conditions. (**a**) Bar plot for the percent of community abundance of the dominant phyla between different feeding sources and temperatures; (**b**) Bar plot for the percent of community abundance of the dominant class between different feeding sources and temperatures; (**c**) Bar plot for the percent of community abundance of the dominant order between different feeding sources and temperatures; (**d**) Bar plot for the percent of community abundance of the dominant family between different feeding sources and temperatures; (**e**) Bar plot for the percent of community abundance of the dominant genus between different feeding sources and temperatures; (**f**) Bar plot for the percent of community abundance of the dominant species between different feeding sources and temperatures. Abbreviations: CM-F: *C. megacephala* fed with wheat bran plus fish; CM-P: *C. megacephala* fed with pig lungs; CM-LT: *C. megacephala* maintained at low temperature; CM-HT: *C. megacephala* maintained at high temperature; CM-VT: *C. megacephala* maintained at variable temperature.

Under all experimental conditions, the dominant phyla were *Proteobacteria* (71.58%), *Firmicutes* (23.68%), *Bacteroidota* (4.36%), and *Actinobacteriota* (0.33%) (Figure 2a and Table S3). The most abundant classes were *Gammaproteobacteria* (52.26%), *Alphaproteobacteria* (19.32%), *Bacilli* (17.26%), *Clostridia* (6.41%), and *Bacteroidia* (4.36%) (Figure 2b and Table S3). The prominent orders included *Cardiobacteriales* (35.88%), *Rickettsiales* (17.80%), *Lactobacillales* (15.13%), *Enterobacterales* (12.55%), and *Peptostreptococcales-Tissierellales* (6.33%) (Figure 2c and Table S3). At the family level, *Wohlfahrtiimonadaceae* (35.88%), *Anaplasmataceae* (17.80%), *Morganellaceae* (10.09%), *Lactobacillaceae* (7.68%), and Family XI (5.64%) were prevalent (Figure 2d and Table S3). Among the top genera bacteria were *Ignatzschineria* (30.01%), *Wolbachia* (17.80%), *Providencia* (8.94%), *Weissella* (6.06%), and *Wohlfahrtiimonas* (5.46%) (Figure 2e, and Table S3). The most representative species included *Ignatzschineria indica* (23.67%), *Wolbachia* spp. (17.80%), *Providencia* spp. (8.94%), *Weissella viridescens* (5.78%), and *Wohlfahrtiimonas chitiniclastica* (5.46%) (Figure 2f and Table S3).

### 3.2. Impact of Diets and Temperatures on the Gut Microbiota Diversity in C. megacephala

The gut bacterial composition of *C. megacephala* exhibited substantial variations under different experimental conditions, as evidenced by the Shannon diversity index (*p*-values < 0.001) (Figure 1b). Comparing the Ace, Chao1, Shannon, and Species observed indices, we found that the gut of *C. megacephala* fed with wheat bran plus fish (CMF) had higher bacterial diversity, compared to specimens fed pig lungs (CMP) (Table 1). Conversely, the group treated with high temperature (35 °C) showed greater bacterial diversity, as indicated by Ace, Chao1, Shannon, and Species observed indices, compared to the other temperature groups (Table 1). Interestingly, beta diversity analysis revealed significant differences both in the gut microbiome of *C. megacephala* between two diets: CMF and CMP, and in the gut microbiome of *C. megacephala* between different temperature groups. The distinction was observed using non-metric multidimensional scaling (NMDS) with binary Jaccard distance. The stress value was 0.166, and the *p*-value was 0.001 (Figure 1c). In addition, both unweighted pair-group method with arithmetic mean (UPGMA) clustering analysis and the heatmap demonstrated significant differences in the gut bacterial community composition of *C. megacephala* across different experimental conditions (Figure S2a,b). The principal coordinate analysis (PCoA) supported this disparity, with PC1 and PC2 explaining 21.89% and 12.33% of variances, respectively (Figure S2c). Furthermore, the partial least squares discriminant analysis (PLS-DA) clearly showed significant dissimilarities not only between CMF and CMP but also within different temperature groups (Figure S2d).

### 3.3. Impact of Different Feeding Sources on Gut Bacterial Communities Across the Lifespan of C. megacephala

The Venn diagrams displayed the presence of 56, 89, and 59 shared OTUs in the gut microbiota of *C. megacephala* at the egg, first instar, and second instar stages, respectively, for both feeding sources (Figure S3a–c). However, *C. megacephala* specimens fed with wheat bran plus fish had 233, 80, and 68 unique OTUs at the egg stage (CMFEggs), first instar larvae (CMFL1), and second instar larvae (CMFL2), respectively. On the other hand, the colony fed with pig lungs produced 177, 72, and 102 unique OTUs at the egg stage (CMPEggs), first larval stage (CMFL1), and second larval stage (CMFL2), respectively (Figure S3a–c). The alpha diversity showed no significant variation in the gut microbiota of *C. megacephala* from the egg to the second larval instar between CMF and CMP (Figure S3g–i). Furthermore, beta diversity through PCA showed significant differences in the gut microbiota composition of *C. megacephala* between CMF and CMP from the egg to the second larval instar. PC1 and PC2 explained 38.4% and 25.8%, 40.32% and 20.13%, and 45.59% and 18.8% of the variances at the egg stage, first larval stage, and second larval stage, respectively (Figure S3m–o).

Significant variations in gut microbiota community composition were observed between different feeding sources from the third larval early stage (L3E) to the adult late stage (AdL). Venn diagrams illustrated the presence of 12, 17, and 19 common core shared OTUs at the third larval instar, pupal stage, and adult stage, respectively (Figure S3d–f). The Shannon and Chao1 alpha diversity indexes indicated significant differences in gut bacterial species richness between different diets at the third larval instar and pupal stage, respectively (Figure S3j,k). However, no significant difference was observed in gut bacterial community richness at the adult stage, as shown by the Ace index (Figure S3l). The beta diversity indexes calculated via PCA and NMDS supported the discrepancy in the gut bacterial communities. In the third larval instar, PCA yielded an R value of 0.8198 (*p*-value = 0.001). PC1 and PC2 explained 24.25% and 13.12% of the variances, respectively (Figure S3p). For the pupal stage, NMDS showed a stress value of 0.106, with R = 0.7811 and *p*-value = 0.001. At the adult stage, NMDS exhibited a stress index of 0.051, with R = 0.8673 and *p*-value = 0.001 (Figure S3q,r).

**Figure 3:**
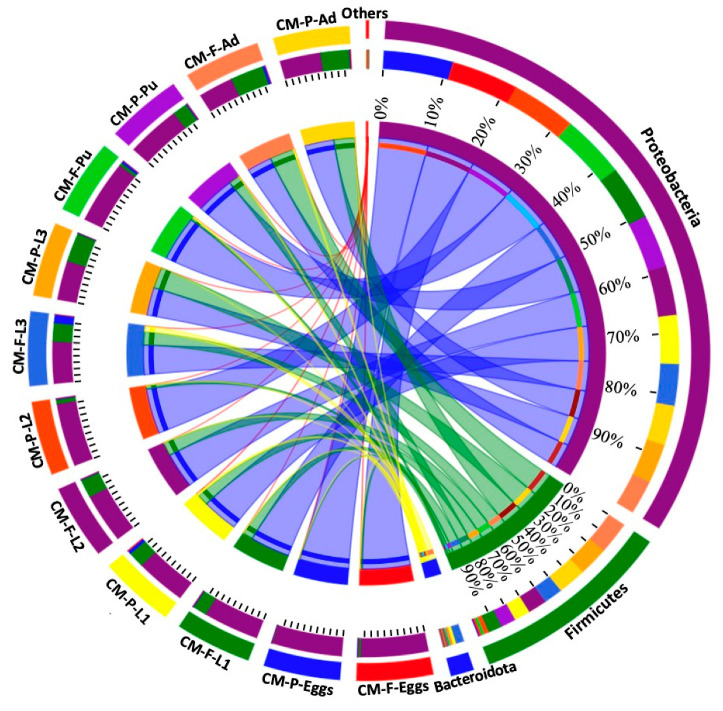
The relative proportions of *Proteobacteria*, *Firmicutes*, and *Bacteroidota* across the lifespan of *C. megacephala* between different feeding sources. The outer and inner circles of the left semicircles symbolize the different developmental stages and the proportion of the species at the phylum level, respectively. The outer and inner circles of the right semicircles indicate the relative abundance at the phylum level and the proportion in different developmental stages groups between different feeding sources. Abbreviations: CM-F: *C. megacephala* fed with wheat bran plus fish; CM-P: *C. megacephala* fed with pig lungs; L1: the first larvae instar of *C. megacephala*; L2: the second larvae instar of *C. megacephala*; L3: the third larvae instar of *C. megacephala*; Pu: the pupae stage of *C. megacephala*; Ad: the adult stage of *C. megacephala*.

At the phylum level, the relative proportions of *Proteobacteria*, *Firmicutes*, and *Bacteroidota* varied according to different feeding sources (Figure 3 and Tables S4–S9). For instance, the *Firmicutes* phylum was more abundant in eggs collected from CMF (3.05%) compared to CMP (0.72%) (Figure 3 and Table S4). Similarly, *Bacteroidota* was more prevalent in CMP (4.65%) than in CMF (0.98%) during the first instar (Figure 3 and Table S5). During the second larvae instar, the *Firmicutes* phylum was more prevalent in CMF (26.80%) than in CMP (6.27%) (Figure 3 and Table S6). For the third larvae stage, the *Bacteroidota* phylum was most dominant during the third larvae late (L3L) and Wandering (WD) stages in CMF (10.54% and 24.90%, respectively) compared to CMP (0.01% and 0.02%, respectively) (Figure 3 and Table S7). During the pupal stages, *Bacteroidota* was most abundant in CMF (6.68%) during the prepupal stage (PP), while the *Firmicutes* phylum was abundant in CMP (20.37%) compared to CMF (1.27%) (Figure 3 and Table S8). During the adult stages, the *Firmicutes* phylum was most prevalent in CMP (54.09%) during the early adult stage (AdE), while at the late stage, it was most abundant in CMF (94.14%) compared to CMP (54.09%) (Figure 3 and Table S9).

**Figure 4:**
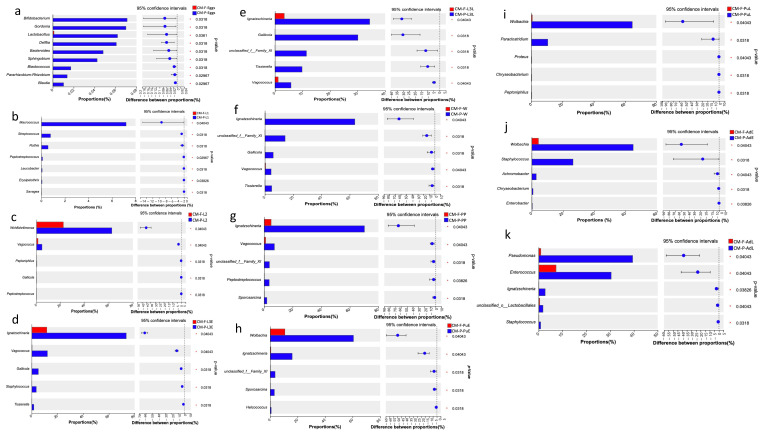
The significant differences in the proportion of bacterial taxa between different feeding sources (Wilcoxon rank-sum, 95% confidence intervals). (**a**) The proportion of bacterial taxa (%) at the egg stage (Eggs) in CMP and CMF; (**b**) The proportion of bacterial taxa (%) at the first larvae instar (L1) in CMP and CMF; (**c**) The proportion of bacterial taxa (%) at the second larvae instar (L2) in CMP and CMF; (**d**–**f**) The proportion of bacterial taxa (%) at the third larvae instar (including L3E, L3L, and Wd stage) in CMP and CMF; (**g**–**i**) The proportion of bacterial taxa (%) at the pupal stage (including PP and PuL) in CMP and CMF; (**j**,**k**) The proportion of bacterial taxa (%) at the adult stage (including AdE and AdL) in CMP and CMF. Abbreviations: CM-F: *C. megacephala* fed with wheat bran plus fish; CM-P: *C. megacephala* fed with pig lungs; L1: the first larvae instar of *C. megacephala*; L2: the second larvae instar of *C. megacephala*; L3E: the third larval early stage of *C. megacephala*; L3L: the third larval late stage of *C. megacephala*; W: Wandering stage of *C. megacephala*; PP: the prepupal stage of *C. megacephala*; PuE: the pupae early stage of *C. megacephala*; PuL: the pupae late stage of *C. megacephala*; AdE: the adult early stage of *C. megacephala;* AdL: the adult late stage of *C. megacephala*.

Comparative analysis using the Wilcoxon rank-sum test, with false discovery rate (FDR) applied at the genus level, revealed significant differences in the proportion of bacterial taxa across different feeding sources. The majority of bacterial taxa displayed higher expression in specimens fed with pork lungs compared to those fed with wheat bran and fish meal (Figure 4). The eggs collected on CMP showed significant differences in the abundance proportions of *Bifidobacterium*, *Gordonia*, *Lactobacillus*, *Delftia*, *Bacteroides*, *Sphingobium*, *Pararhizobium-Rhizoobium*, and *Blautia* compared to CMF (Figure 4a). At the first larvae instar (L1), the abundance proportions of *Macrococcus*, *Streptococcus*, *Rothia*, *Peptostreptococcus*, *Leucobacter*, *Erysipelothrix*, and *Savagea* were significantly higher in CMP than in CMF (Figure 4b). At the second larvae instar (L2), *Wohlfahrtiimonas*, *Vagococcus*, *Peptoniphilus*, *Gallicola*, and *Peptostreptococcus* were significantly more predominant in CMP than in CMF (Figure 4c).

During the third larvae instars, including L3E, L3L, and Wd stages, *Ignatzschineria*, *Vagococcus*, *Gallicola*, and *Tissierella* were the most dominant in CMP compared to CMF (Figure 4d–f). From the prepupal stage (PP) to the pupae late stage (PuL), there were significantly higher proportions and abundances of *Ignatzschineria*, *Vagococcus*, *Peptostreptococcus*, *Sporosarcina*, *Wolbachia*, *Helcococcus*, *Proteus*, *Paraclostridium*, and *Chryseobacterium* in CMP compared to CMF (Figure 4g–i). During the adult stage, *Wolbachia*, *Staphylococcus*, *Achromobacter*, *Chryseobacterium*, *Enterobacter*, *Pseudomonas*, *Enterococcus*, *Ignatzschineria*, and *Staphylococcus* were significantly more dominant in CMP than in CMF (Figure 4j,k).

### 3.4. Impact of Different Temperature Conditions on Gut Bacterial Communities Across the Lifespan of C. megacephala

In the third larval stage, there was a significant difference in the composition of the gut microbiome between different temperature conditions (*p* = 0.006). This was indicated by the alpha diversity measured through the Shannon index (Figure S4a). The Chao1 index also showed considerable variation in the gut microbiome of *C. megacephala* at the pupal stage under different temperature treatments (*p* = 0.003) (Figure S4b). Furthermore, during the adult stage, there were significant variations in the gut microbiome community under different temperature conditions, as demonstrated by the Shannon index (*p* < 0.001) (Figure S4c). Additionally, the PCoA analysis demonstrated that the gut bacterial communities of the four temperature conditions (LT, MT, HT, and VT) were significantly different from the third larval stage to the adult stage (Figure S4d–f).

**Figure 5:**
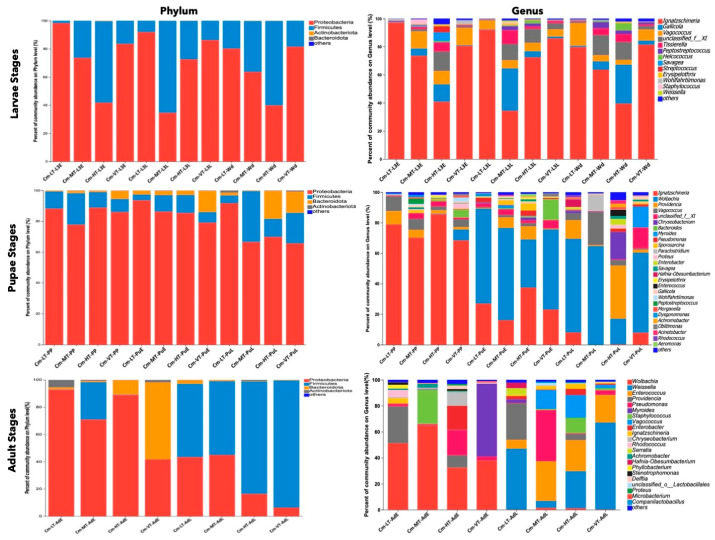
The dominant gut microbiota under different temperature conditions among the developmental stages. The diagrams on the first row display the percent of community abundance (%) at the third larvae instar on phylum and genus level. The diagrams on the second row display the percent of community abundance (%) at the pupal stage on phylum and genus level. The diagrams on the last row display the percent of community abundance (%) at the adult stage on phylum and genus level. Abbreviations: CM-LT: *C. megacephala* maintained at low temperature; CM-MT: *C. megacephala* maintained at moderate temperature; CM-HT: *C. megacephala* maintained at high temperature; CM-VT: *C. megacephala* maintained at variable temperature; L3E: the third larval early stage of *C. megacephala*; L3L: the third larval late stage of *C. megacephala*; Wd: Wandering stage of *C. megacephala*; PP: the prepupal stage of *C. megacephala*; PuE: the pupae early stage of *C. megacephala*; PuL: the pupae late stage of *C. megacephala*; AdE: the adult early stage of *C. megacephala;* AdL: the adult late stage of *C. megacephala*.

At the phylum level, *Proteobacteria* (69.53%), *Firmicutes* (25.65%), *Bacteroidota* (4.37%), and *Actinobacteriota* (0.42%) were the dominant phyla found in the gut microbiome of *C. megacephala* across different temperature conditions, from the third larval stage to adult stages (Table S10). The proportions of these phyla varied among the developmental stages and temperature conditions (Figure 5, Tables S10–S15). During the third larval stage, the abundance of *Proteobacteria* decreased as the temperature increased from 15 to 35 °C, while the abundance of *Firmicutes* increased with higher temperatures (Figure 5, Table S12). During the pupal stage, the phylum *Bacteroidota* showed higher abundance in the VT group from the PP to PuL stages, compared to the other temperature groups. Additionally, a higher proportion of *Bacteroidota* was found in the PuL stage, compared to other temperature groups (Figure 5, Table S13). In the early adult stage, *Proteobacteria* dominated in the LT, MT, and HT groups, while *Bacteroidota* was dominant in the VT group. The *Firmicutes* phylum had higher abundance in the MT group compared to other groups. During the late adult stage, *Firmicutes* were most abundant, with higher proportions found in the HT and VT groups (Figure 5, Table S14).

At the genus level, Venn diagrams revealed 12, 26, and 21 common core bacterial genera in the larvae, pupae, and adult stages, respectively (Figure S4g–i). The most prevalent bacteria in the larval stages were *Ignatzschineria* (70.06%), *Gallicolas* (7.60%), *Vagococcus* (7.47%), unclassified Family_XI (6.04%), and *Tissierella* (2.81%) (Figure 5 and Table S11). In the pupal stage, the common core bacteria included *Ignatzschineria* (36.24%), *Wolbachia* (35.07%), *Vagococcus* (5.06%), *Providencia* (4.44%), and unclassified Family_XI (3.12%) (Figure 5 and Table S11). The dominant genera in the adult stage were *Wolbachia* (23.73%), *Weissella* (18.39%), *Enterococcus* (10.31%), *Providencia* (8.87%), and *Pseudomonas* (8.68%) (Figure 5 and Table S11).

**Figure 6:**
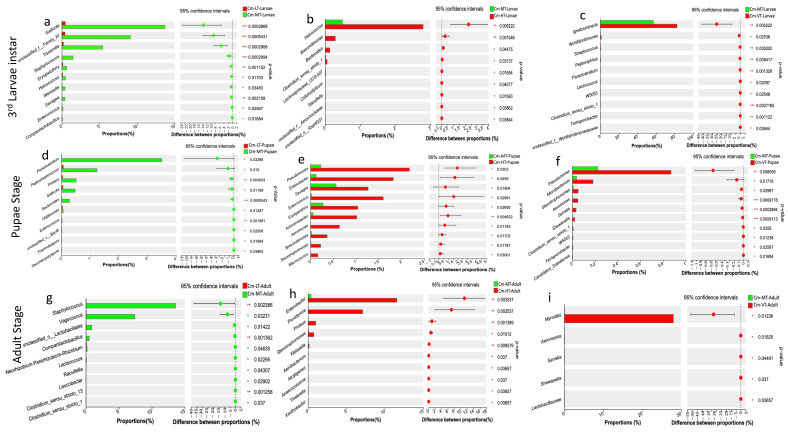
Comparative analysis revealed significant differences in the proportion of bacterial taxa across different temperatures (Wilcoxon rank-sum, 95% confidence intervals). (**a**–**c**) The proportion of bacterial taxa (%) at the third larvae instar with the low, high and variable temperatures compared to the moderate temperature, respectively; (**d**–**f**) The proportion of bacterial taxa (%) at the pupal stage with the low, high and variable temperatures compared to the moderate temperature, respectively; (**g**–**i**) The proportion of bacterial taxa (%) at the adult stage with the low, high and variable temperatures compared to the moderate temperature, respectively. Abbreviations: CM-LT: *C. megacephala* maintained at low temperature; CM-MT: *C. megacephala* maintained at moderate temperature; CM-HT: *C. megacephala* maintained at high temperature; CM-VT: *C. megacephala* maintained at variable temperature.

The Wilcoxon rank-sum test analysis revealed that the low temperature condition decreased the abundance of certain bacterial taxa, while the hot and variable temperature treatments increased the abundance of specific bacterial colonies. In the larval stage, the low temperature treatment significantly reduced the abundance of *Gallicola*, unclassified_family_XI, *Tissierella*, *Staphylococcus*, *Erysipelothrix*, *Helcococcus*, *Weissella*, *Savagea*, and *Enterococcus*. In the pupal stage, it reduced the abundance of *Paraclostridium*, *Peptostreptococcus*, *Proteus*, *Gallicola*, *Bacteroides*, *Oblitimonas*, and *Enterococcus*. In the adult stage, it reduced the abundance of *Staphylococcus*, *Vagococcus*, unclassified_family_XI, and *Companilactobacillus* (Figure 6a,d,g). From the larval to adult stages, the hot temperature condition increased the relative abundance of *Helcococcus*, *Brevundimonas*, *Bacteroides*, *Clostridium_sensu_stricto_1*, *Pseudomonas*, *Enterobacter*, *Savagea*, *Enterococcus*, *Erysipelothrix*, *Achromobacter*, *Aeromonas*, *Stenotrophomonas*, *Macrococcus*, *Enterobacter*, *Providencia*, *Proteus*, and *Klebsiella* (Figure 6b,e,h). Conversely, the variable temperature condition reduced the relative abundance of *Ignatzschineria*, *Wohlfahrtiimonas*, *Streptococcus*, *Peptoniphilus*, *Paraclostridium*, *Lactococcus*, *Pseudomonas*, *Microbacterium*, *Stenotrophomonas*, *Aeromonas*, *Serratia*, *Shewanella*, and *Myroides* from the larval to adult stages (Figure 6c,f,i).

### 3.5. Functional Prediction and the Phenotype Composition of the Gut Microbiome of C. megacephala

**Figure 7:**
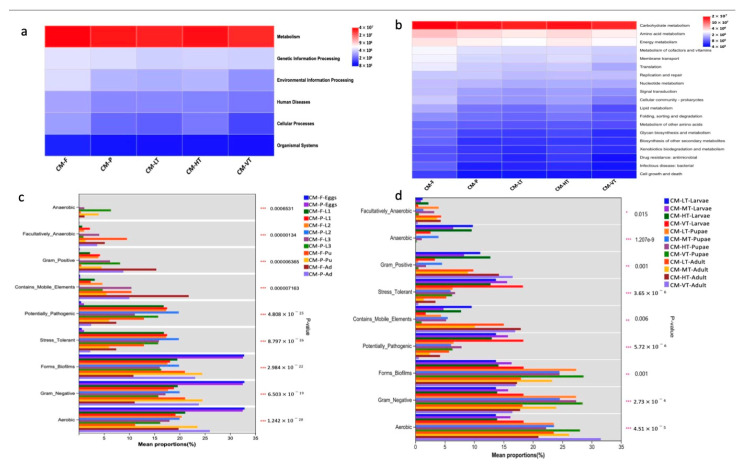
The KEGG functional prediction and the phenotype composition of the gut microbiome of *C. megacephala*. (**a**,**b**) Heatmaps at the KEGG functional prediction pathway level 1 and level 2. Red, blue and white colors denote marked positive, negative and no significant result, respectively; (**c**,**d**) The phenotype composition of the gut microbiome of *C. megacephala* (mean proportion), which was significantly influenced by different feeding sources and temperature conditions (*** *p* < 0.001). Abbreviations: CM-F: *C. megacephala* fed with wheat bran plus fish; CM-P: *C. megacephala* fed with pig lungs; CM-LT: *C. megacephala* maintained at low temperature; CM-MT: *C. megacephala* maintained at moderate temperature; CM-HT: *C. megacephala* maintained at high temperature; CM-VT: *C. megacephala* maintained at variable temperature; L1: the first larvae instar of *C. megacephala*; L2: the second larvae instar of *C. megacephala*; L3: the third larvae instar of *C. megacephala*; Pu: the pupae stage of *C. megacephala*; Ad: the adult stage of *C. megacephala*.

The KEGG functional prediction pathways were visualized as heatmaps at two hierarchical levels (Figure 7a,b). At pathway level 1, the gut microbiome of *C. megacephala* was mainly dominated by metabolism function under all experimental conditions (Figure 7a). At pathway level 2, twenty pathways were prevalent, with carbohydrate metabolism being the most dominant overall, followed by amino acid metabolism (Figure 7b). The phenotype composition of the gut microbiome of *C. megacephala* exhibited various characteristics, including aerobic, anaerobic, facultatively anaerobic, biofilm-forming, gram-negative, gram-positive, containing mobile elements, stress-tolerant, and potentially pathogenic bacteria (Figure 7c,d and Table S16). The phenotype composition of the gut microbiome of *C. megacephala* was significantly influenced by different feeding sources and temperature conditions (Figure 7c,d and Table S16).

## 4. Discussion

The present study investigates the impact of different feeding sources and temperature variations on the diversity and composition of the gut microbiome of *C. megacephala* throughout its life cycle. The study utilizes 16S rRNA gene sequencing to examine the configuration and heterogeneity of the gut microbiome. Results demonstrate a wide range of bacteria in the gut, indicating a symbiotic relationship with the host. Analysis of alpha and beta diversity indexes reveal significant variations in the gut bacterial community of *C. megacephala* under different feeding sources and temperature treatments. This is consistent with previous studies that suggest diet and habitat conditions can influence gastrointestinal bacteria in insects [30,34,81]. Overall, under experimental conditions, the gut bacterial colonies in *C. megacephala* were dominated by the phyla *Proteobacteria* (71.58%), *Firmicutes* (23.68%), and *Bacteroidota* (4.36%) (Figure 2a and Table S3), as previously reported [27,30,31]. Similar findings have been reported in the gut of other insects, such as *M. domestica* [82,83], *Chironomus riparius* (Diptera: *Chironomidae)* [84], *P. fuscipes* [15], *Copris incertus* (Coleoptera: *Scarabaeidae*) [85], *H. illucens* [86], and *C. grahami* (Diptera: *Calliphoridae*) [41]. Our findings are consistent with those reported by Junqueira et al. [12], who stated that *Proteobacteria*, *Firmicutes*, and *Bacteroidota* were the most dominant phyla of bacteria in the gut microbiome of species belonging to the *Calliphoridae* and *Muscidae* families. During their investigation on the effects of diets on the gut microbiota of *C. grahami* throughout its lifespan, Li et al. [41] found that *Firmicutes*, *Proteobacteria*, and *Bacteroidetes* were the most dominant phyla identified. Singh et al. [29] observed that blowfly species such as *L. sericata* and *L. cuprina*, when fed beef liver, displayed a prevailing dominance of the phyla *Proteobacteria* and *Firmicutes* in their gut microbiome.

According to the phylogenetic tree and bar plots of gut bacterial communities at the genus level, certain genera were found to be highly dominant in the gut microbiome of *C. megacephala* throughout its life stages, regardless of the food source (Figure S5). Specifically, the most dominant genera were *Wolbachia*, *Wohlfahrtiimonas*, *Ignatzschineria*, *Providencia*, *Pseudomonas*, *Acetobacter*, and *Morganella*, which belong to the phylum *Proteobacteria*. Additionally, the genera *Weissella*, *Vagococcus*, *Gallicola*, *Enterococcus*, and *Staphylococcus*, from the phylum *Firmicutes*, as well as *Dysgonomonas* from the phylum *Bacteroidota*, were also found to be prevalent in the gut microbiome of *C. megacephala* from the eggs to the adult stages. Our result corroborated those found by Wang et al. [30], who also identified *Ignatzschineria*, *Vagococcus*, *Wohlfahrtiimonas*, *Providencia*, *Pseudomonas*, *Enterococcus*, *Staphylococcus*, and *Dysgonomonas* in the gut microbiota of *C. megacephala* across its entire life cycle at the genus level. In addition, *Ignatzschineria*, *Providencia*, and *Pseudomonas* have been identified in the larvae of *C. megacephala* in the presence of manure microbiome, heavy metal stability and greenhouse gas emissions [27]. These bacterial colonies have been identified as the most common genera in the gut microbiomes of *Calliphoridae* and Muscidae species [12,29,87,88]. Therefore, they are considered as the predominant bacteria in the gut of fly species [41].

Our findings revealed that the alpha diversity of the gut bacterial community in eggs, first instar larvae, second instar larvae, and adult stages of *C. megacephala* were not significantly affected by different feeding sources (Figure S3g–i,l). However, significant variations in the alpha diversity Shannon and Chao1 indexes were observed between CMF and CMP during the third instar larvae and pupal stages of *C. megacephala*. Furthermore, beta diversity, as determined by PCA and NMDS analysis, showed differences in the gut bacterial colonies obtained from each feeding source, indicating variations in bacterial communities based on feeding sources and developmental stages (Figure S3m–r). The findings suggest that the quality of nutrients and species consumption at each stage of development has a more significant impact on the gut microbiome of *C. megacephala*, compared to the feeding sources. However, the eggs, immature larvae, and adult stages of *C. megacephala* exhibited a consistent species richness and community structure of gut bacteria even when exposed to different feeding sources. This aligns with previous research on the gut microbiota of other insect species, such as *C. grahami* [41], *H. illucens* [89], and *Periplaneta americana* [90] which also showed no significant variations in the gut bacterial community in response to different feeding sources [41]. In contrast, distinct feeding sources have been observed to impact the gut microbiome of other insects, including *Lymantria dispar* [91], *Helicoverpa armigera* [92], *Drosophila melanogaster* [93], and *Adelphocoris suturalis* [94]. The diverse effects of different food sources on the gut microbiome of insects indicate that insects residing in low-bacterial habitats are particularly susceptible to environmental factors which can impact their gut bacterial communities. In contrast, insects living in complex bacterial environments generally exhibit more stable compositions in their gut microbiomes [41].

*Proteobacteria*, a potential indicator of dysbiosis and antibiotic resistance genes in the guts of soil invertebrates [15,95], was most dominant from the eggs to the early adult stage in both feeding sources. However, in the late stage of the third larvae, *Proteobacteria* was more dominant in CMF than in CMP, and in the late adult stage, it was more abundant in CMP than in CMF. The phylum *Firmicutes* was abundant during the pupal late stage in CMP and during the adult late stage in both feeding sources. The proportion of *Firmicutes* was higher in the CMF than in the CMP.

Ambient temperatures have been shown to significantly impact the biology of poikilothermic organisms, such as Diptera [96]. In our study, the alpha and beta diversity estimators indicated a significant effect of different temperature treatments on the gut microbiome of *C. megacephala* (Figure S4a–f). We observed significant variations in the gut bacterial composition and community structure across the developmental stages, from the third instar larvae to adult. These findings are consistent with previous studies conducted by Wang et al. [30], who also observed significant variations in the gut bacterial community and composition structure from the immature to adult stages of *C. megacephala*. At the phylum stage, the proportion abundance of *Proteobacteria* decreased with increasing temperatures during the larval stages. Meanwhile, the *Firmicutes* phylum was the most abundant in the HT group compared to the other groups. During the pupal stages, the phylum *Proteobacteria* was the most dominant across all temperature conditions. However, a higher proportion of the phylum *Bacteroidota* was found in the VT group, compared to the other groups. As for the adult stage, *Proteobacteria* dominated during the early adult stage in the LT, MT, and HT groups, while *Bacteroidota* dominated in the VT group. In the late adult stage, *Firmicutes* were the most abundant with higher proportions in the HT and VT groups (Figure 5). These results indicate that the proportions and abundances of bacterial colonies in *C. megacephala* were significantly influenced by the different temperature treatments.

However, when examining the Venn diagrams at the genus levels, it was observed that there were 12, 26, and 21 bacteria commonly shared between the third larval instar, pupal stage, and adult stage, respectively, across all temperature conditions (Figure S4g–i). Among these commonly shared bacteria, the most representative were *Ignatzschineria*, *Gallicola*, *and Vagococcus* during the larval stages; *Ignatzschineria*, *Wolbachia*, *Providencia*, and *Vagococcus* during the pupal stages; and *Wolbachia*, *Weissella*, *Enterococcus*, *Providencia*, *Pseudomonas*, *Myroides*, *Staphylococcus*, *Vagococcus*, *Enterobacter*, and *Ignatzschineria* during the adult stages (Figure 5). These bacteria are often identified in the gut microbiomes of several insects and are considered as common core bacteria [29,41,97]. This suggests that there is a certain level of stability in the gut bacterial colonies of this scavenging species. Temperature treatment had diverse effects on the gut microbiome of *C. megacephala* throughout its life cycle (Figure 6). In our study, the low temperature condition (15 °C) negatively affected the abundance of several bacterial taxa during the development stages of *C. megacephala* consistent with the previous studies [98]. The abundance of bacterial community colonies such as *Gallicola*, *Tissierella*, *Paraclostridium*, *Peptostreptococcus*, *Staphylococcus*, and *Vagococcus* significantly decreased from the third larval instar to the adult stages in *C. megacephala* (Figure 6a,d,g). On the other hand, the high temperature condition (35 °C) and the variable temperature treatment (average of 23.31 °C) positively influenced the abundance of several bacterial communities throughout the lifespan of *C. megacephala*. The high temperature condition stimulated a significant increase in the abundance of *Helcococcus*, *Brevundimonas*, *Pseudomonas*, *Enterobacter*, *Savagea*, *Enterococcus*, *Erysipelothrix*, *Achromobacter*, *Enterobacter*, and *Providencia* from the third larval instar to the adult stages in *C. megacephala* (Figure 6b,e,h). Additionally, the variable temperature treatment (average of 23.31 °C) favored an increase in the prevalence of *Ignatzschineria*, *Pseudomonas*, *Microbacterium*, and *Myroides* from the third larval instar to the adult stages in *C. megacephala* (Figure 6c,f,i). These diverse effects align with seasonal variations in bacterial communities of green bottle flies, where *Staphylococcus* was predominant in spring, *Ignatzschineria* in summer, and *Vagococcus*, *Dysgonomonas*, and an unclassified *Acetobacteraceae* in autumn [99]. *Drosophila melanogaster* has shown a high prevalence of *Wolbachia* and *Acetobacter* at low and high temperatures, respectively [100]. *Lactobacillus plantarum* and *Corynebacterium nuriki* have been identified to play crucial roles in determining the thermal preference of *D. melanogaster* [101]. These findings suggest that fluctuations in the host’s environment can impact gut bacteria abundance [99,102]. Additionally, it has been observed that fluctuations in the host’s environment can impact the abundance of gut bacteria, thereby affecting the host’s thermophysiology [99,102].

In our study, *C. megacephala* exhibited a high abundance of *Proteobacteria*, *Firmicutes*, and *Bacteroidota* from the third larval stage to adult stages, which was influenced by temperature. The *Proteobacteria*, *Firmicutes*, and *Bacteroidota* phyla play vital roles in various physiological processes, such as nutrition provision, digestion, excretion, reproduction, immunity, and communication [13,15,103]. These phyla are highly abundant in the gut microbiome of *C. megacephala*, suggesting a potential association with metabolic functions, particularly carbohydrate and amino acid metabolisms (Figure 7a,b). Similar functional predictions were observed in the gut microbiota of *C. grahami* [41], indicating that the 16S rRNA gene predictions in these Diptera species are primarily related to metabolism. At the genus level, *Ignatzschineria* has been associated with chitin degradation and metamorphosis [29,104]. *Wolbachia* is associated with functions related to energy production, translation, and cellular processes [105]. *Providencia* provides nutrients and aids in the degradation of xylan [29,106]; therefore, it plays a significant role in pupal maturation and adult emergence in extreme temperature environments [86].

Moreover, the phenotypic characteristics of the gut microbiota in *C. megacephala* under all experimental conditions include aerobic and facultative anaerobic gram-negative and gram-positive bacteria, biofilm formers, stress-tolerant bacteria, potentially pathogenic bacteria, bacteria containing mobile elements, and anaerobic bacteria (Figure 5). The gut microbiome of *C. megacephala* harbors potentially pathogenic bacterial species, such as *Ignatzschineria indica* and *Ignatzschineria ureiclastica*, which are associated with human infections, including sepsis [107,108] and bacteremia [109,110]. *Wolbachia* spp., on the other hand, are mutualistic in *Onchocercidae* species, causing human filarial diseases [111]. *Weissella viridescens* is capable of thriving in both variable and low temperatures. It is frequently encountered in the context of meat production under low temperature conditions [112]. Some species remained unclassified due to limitations in the 16S rRNA gene sequencing resolution [21]. However, prior studies have detected *I. indica*, and *I. ureiclastica* in the *C. megacephala* [30,31], highlighting their medical, veterinary, economic, and commercial importance.

## 5. Conclusions

This study sought to examine the effects of diverse feeding sources and temperature variations on the gut microbiome of *Chrysomya megacephala* through the application of 16S rRNA gene sequencing. The results demonstrated a broad spectrum of bacterial taxa present in the gut of *C. megacephala*, with varying levels of abundance contingent upon the feeding sources and temperature conditions. While the diversity and structural composition of the gut microbiome were notably influenced by these factors, the overall community composition exhibited relative stability. These findings underscore the potential risks and benefits associated with the diverse bacterial species inhabiting the gut of *C. megacephala*. Further investigation is warranted to pinpoint the specific genes that govern the dietary and thermal preferences of these bacteria and to elucidate their functional roles throughout the lifespan of *C. megacephala*.

## Figures and Tables

**Table 1:** Richness and diversity estimate overall temperature conditions based on the 16S rRNA gene libraries from the sequencing analysis.

Estimators/Temperatures *	CM-F	CM-P	LT	HT	VT	*p*-Values
Ace index	126.8	117.4	86.54	149.1	103.2	1.56 × 10^−5^
Chao1 index	122.6	108.1	79.19	127.2	92.92	4.30 × 10^−5^
Coverage	0.9992	0.9992	0.9994	0.999	0.9993	8.30 × 10^−4^
Shannon index	1.82	1.362	0.9303	1.794	1.223	3.86 × 10^−7^
Simpson	0.344	0.4554	0.5886	0.3133	0.4637	2.26 × 10^−5^
Species Observed (Sobs)	104.3	83.55	58.67	88.09	70.21	3.01 × 10^−6^

* CM-F and CM-P correspond to *C. megacephala* fed with fish meal and pork lungs, respectively, while LT, HT, and VT represent low, hot, and variable temperatures, respectively.

## Data Availability

The original contributions presented in this study are included in the article/Supplementary Material. Further inquiries can be directed to the corresponding authors.

## Supplementary Materials

The following supporting information can be downloaded at: https://www.mdpi.com/article/10.3390/insects16030283/s1, Figure S1: The analysis of rank abundance showed by Shannon curves indicated sufficient sampling; Figure S2: The gut bacterial composition of *C. megacephala* exhibited substantial variations under different experimental conditions; Figure S3: The variations in the proportion of bacterial taxa across the lifespan of *C. megacephala* between different feeding sources; Figure S4: The variations in the proportion of bacterial taxa across the lifespan of *C. megacephala* under different temperature conditions; Figure S5: Phylogenetic tree showed the gut bacterial communities on the genus level; Table S1: Statistical sequencing information across the life history of *C. megacephala* at different feeding sources; Table S2: Statistical sequencing information across the life history of *C. megacephala* at different temperatures; Table S3: Top five bacteria identified at different taxonomic levels in the gut microbiome of *C. megacephala* across all experimental conditions; Table S4: Top five bacteria identified at different taxonomic levels in the eggs of *C. megacephala* collected on different feeding sources; Table S5: Top five bacteria identified at different taxonomic levels during the first instar larvae (L1) of *C. megacephala* collected on different feeding sources; Table S6: Top five bacteria identified at different taxonomic levels during the first instar larvae (L1) of *C. megacephala* collected on different feeding sources; Table S7: Top five bacteria identified at different taxonomic levels during the third instar larvae (L3) of *C. megacephala* collected on different feeding sources; Table S8: Top five bacteria identified at different taxonomic levels during the pupal stages of *C. megacephala* collected on different feeding sources; Table S9: Top five bacteria identified at different taxonomic levels during the adult stages of *C. megacephala* collected on different feeding sources; Table S10: Top five bacteria identified at different taxonomic levels in the gut microbiome of *C. megacephala* across all temperature conditions; Table S11: Top five bacteria identified at different taxonomic levels during the development stages of *C. megacephala* under overall temperature conditions; Table S12: Top five bacteria identified at different taxonomic levels during the third larval stages of *C. megacephala*, reared under different constant and variable temperatures; Table S13: Top five bacteria identified at different taxonomic levels during the pupal stages of *C. megacephala*, raised at different constant and variable temperature; Table S14: Top five bacteria found at different taxonomic levels during the adult stages of *C. megacephala*, reared under different constant and variable temperatures; Table S15: Global view of top five bacteria identified at different taxonomic levels in response to different temperature treatments on the gut of *C. megacephala*; Table S16: Phenotype of top five bacterial genera at different development stages of *C. megacephala* raised at different constant and variable temperatures.

## Abbreviations

The following abbreviations are used in this manuscript:

*C. megacephala*	*Chrysomya megacephala*
RH	Relative humidity
L/D	Light-dark
OTU	Operational Taxonomic Unit
LT	Low Temperature
HT	High Temperature
MT	Moderate Temperature
VT	Variable Temperature
CM-F	*C. megacephala* fed with wheat bran plus fish
CM-P	*C. megacephala* fed with pig lungs
