# Supplementary material for: Effects of Feeding Sources and Different Temperature Changes on the Gut Microbiome Structure of Chrysomya megacephala (Diptera: Calliphoridae)
Source: Insects. 2025 Mar 8;16(3):283. doi: 10.3390/insects16030283 (PMC11943086; doi:10.3390/insects16030283)
Supplement: Supplementary file 1 [file insects-16-00283-s001.zip › insects-3434865-supplementary/supplementary files/Figure S1-S5.pdf]

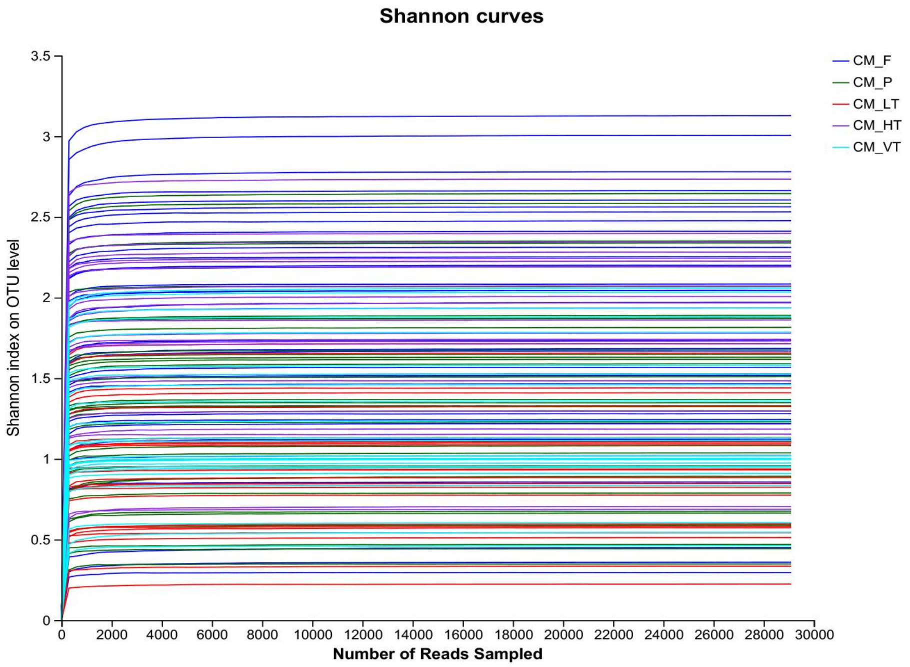

Fig.S1.The analysis of rank abundance showed by Shannon curves indicated sufficient sampling.

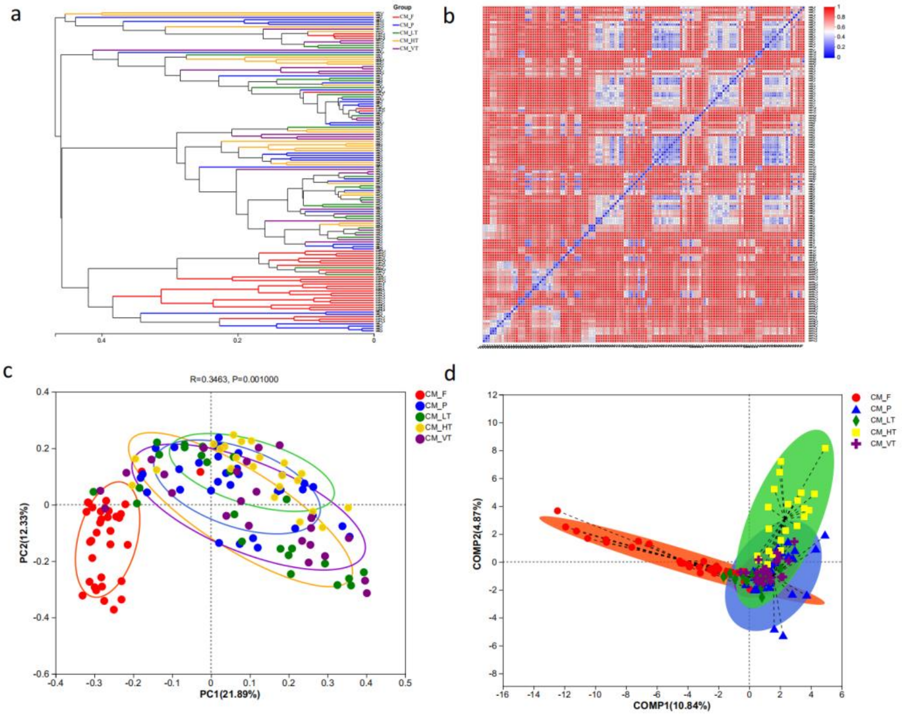

Fig.S2.The gut bacterial composition of *C.megacephala* exhibited substantial variations under different experimental conditions. a)Unweighted pair-group method with arithmetic mean (UPGMA) clustering analysis about the gut bacterial community composition of *C.megacephala* across different experimental conditions. **b)**Heatmap about the gut bacterial community composition of *C.megacephala* across different experimental conditions. Red, blue and white colors denote marked positive, negative and no significant result, respectively. **c)**The principal coordinate analysis (PCoA). PC1 and PC2 showed 21.89% and 12.33% of variances, respectively. **d)**The partial least squares discriminant analysis (PLS-DA) showed the differences about the gut bacterial community composition of *C. megacephala* across different experimental conditions.

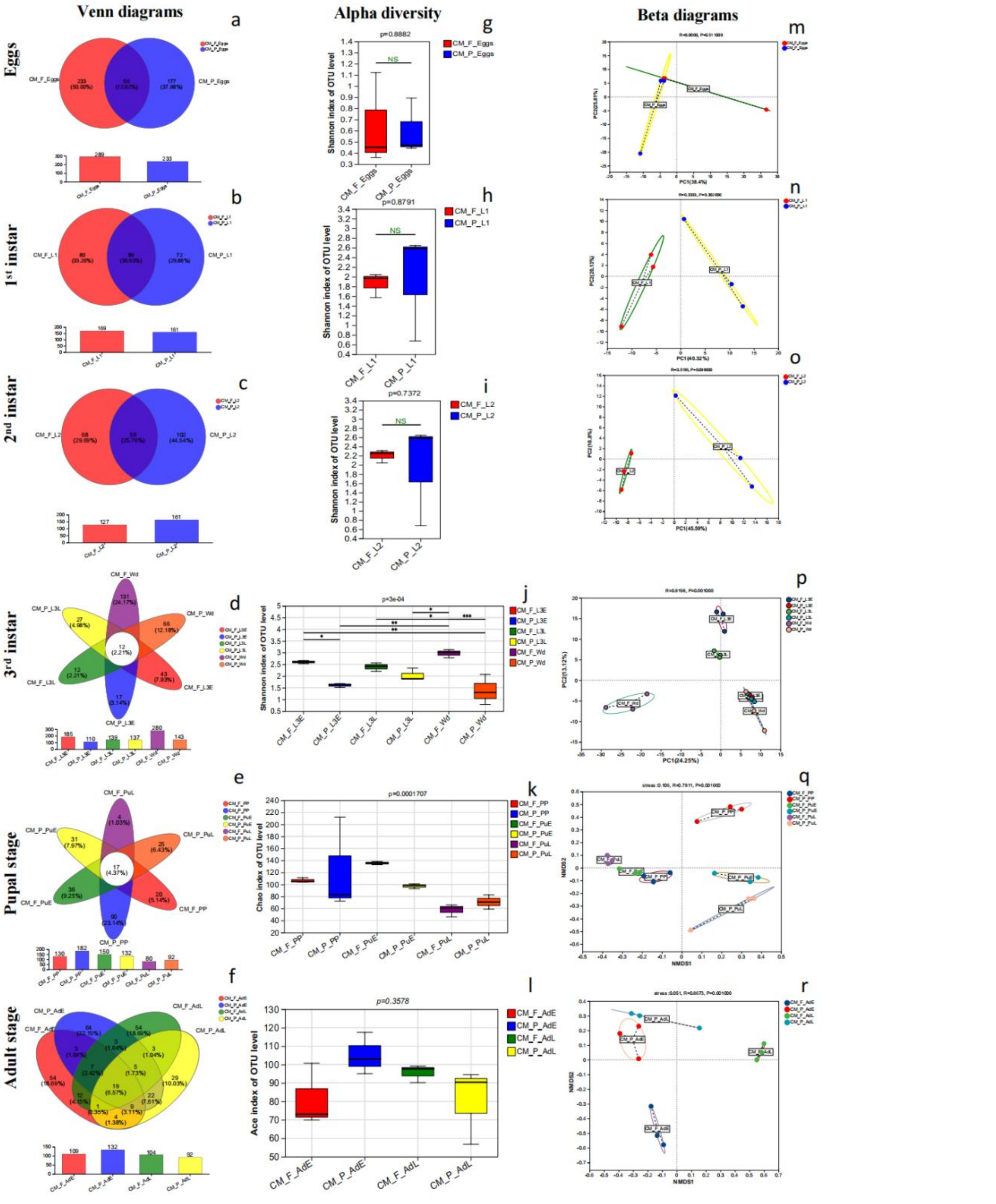

Fig.S3.The variations in the proportion of bacterial taxa across the lifespan of C. megacephala between different feeding sources. a-f)Venn diagrams displayed the OTUs shared by CM-F and CM-P during the egg, first instar, second instar, third instar, pupal, and adult stages. g-l)Alpha diversity analysis across the lifespan of C. megacephala between different feeding sources(*p<0.05, **P<0.01, ***p<0.001). m-r)Beta diversity analysis across the lifespan of C. megacephala between different feeding sources.

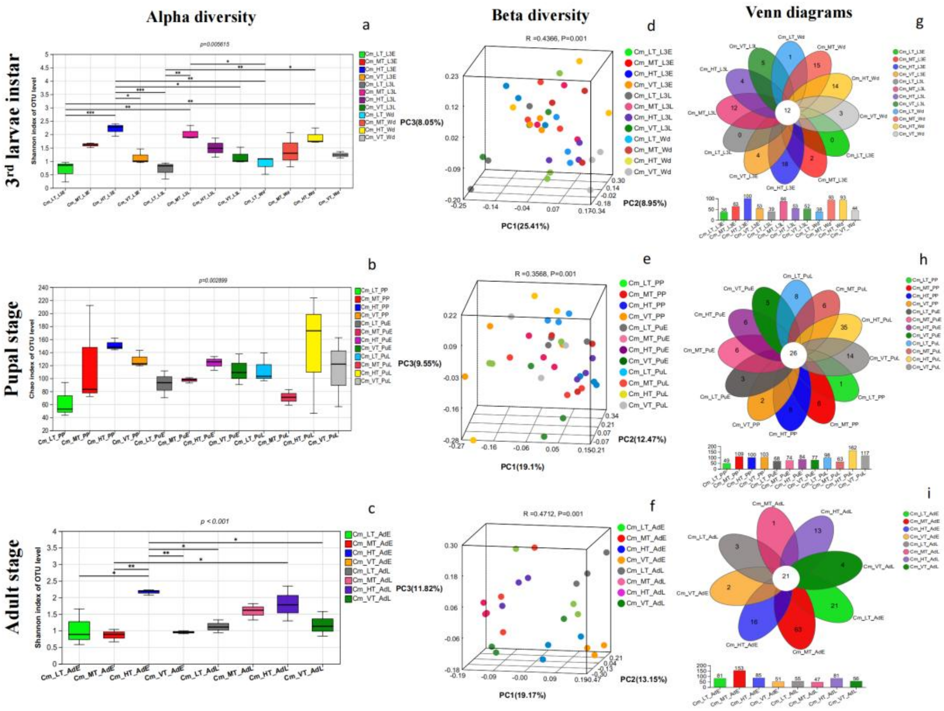

Fig.S4.The variations in the proportion of bacterial taxa across the lifespan of C. megacephala under different temperature conditions. a-c)Alpha diversity analysis displayed the differences during the third instar, pupal, and adult stages under different temperature conditions.(*p<0.05, **P<0.01, ***p<0.001). d-f)Beta diversity analysis during the third instar, pupal, and adult stages under different temperature conditions. g-i)Venn diagrams showed the OTUs shared by LT, MT, HT, and VT groups during the third instar, pupal, and adult stages.

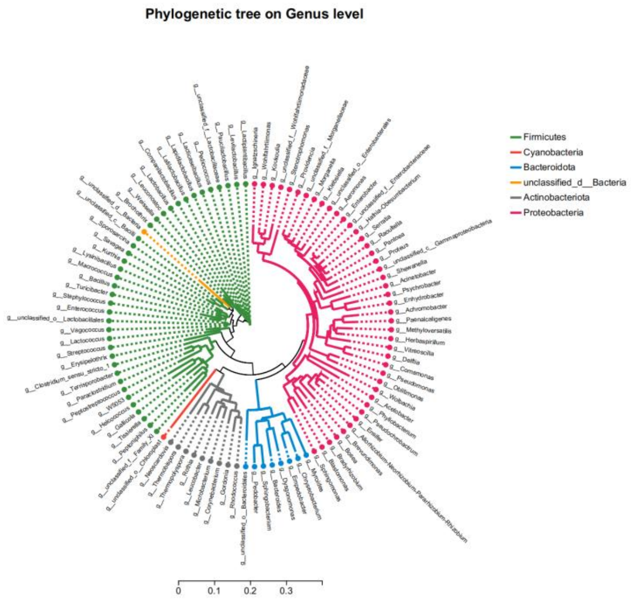

Fig.S4.Phylogenetic tree showed the gut bacterial communities on the genus level.
