# Supplementary material for: Effects of Feeding Sources and Different Temperature Changes on the Gut Microbiome Structure of Chrysomya megacephala (Diptera: Calliphoridae)
Source: Insects. 2025 Mar 8;16(3):283. doi: 10.3390/insects16030283 (PMC11943086; doi:10.3390/insects16030283)
Supplement: Supplementary file 1 [file insects-16-00283-s001.zip › insects-3434865-supplementary/supplementary files/Figure S3.pdf]

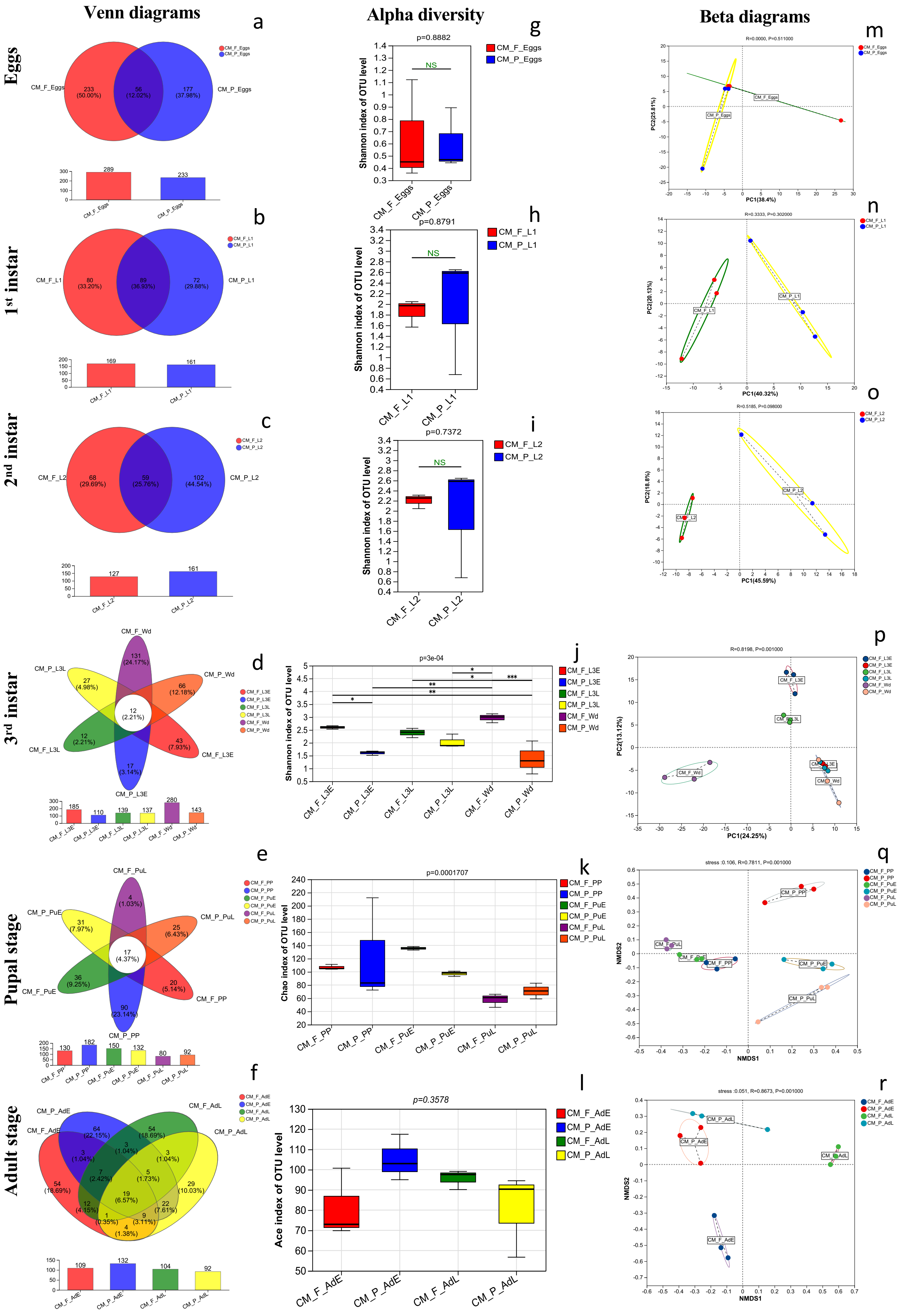

Venn diagrams
Alpha diversity
Beta diagrams
Eggs
1st instar
2nd instar
3rd instar
Pupal stage
Adult stage
a
b
c
d
e
f
g
h
i
j
k
l
m
n
o
p
q
r
CM_F_Eggs
CM_P_Eggs
233 (50.00%)
56 (12.02%)
177 (37.98%)
289
233
CM_F_L1
CM_P_L1
80 (33.20%)
89 (36.93%)
72 (29.88%)
169
161
CM_F_L2
CM_P_L2
68 (29.69%)
59 (25.76%)
102 (44.54%)
127
161
CM_F_Wd
CM_P_Wd
CM_P_L3L
CM_F_L3L
CM_F_L3E
CM_P_L3E
131 (24.17%)
27 (4.98%)
66 (12.18%)
12 (2.21%)
12 (2.21%)
43 (7.93%)
17 (3.14%)
185
110
139
137
280
143
CM_F_PuL
CM_P_PuE
CM_P_PuL
CM_F_PuE
CM_F_PP
CM_P_PP
4 (1.03%)
31 (7.97%)
25 (6.43%)
17 (4.37%)
36 (9.25%)
20 (5.14%)
90 (23.14%)
130
182
150
132
80
92
CM_P_AdE
CM_F_AdE
CM_F_AdL
CM_P_AdL
64 (22.15%)
54 (18.69%)
3 (1.04%)
3 (1.04%)
3 (1.04%)
7 (2.42%)
5 (1.73%)
54 (18.69%)
29 (10.03%)
19 (6.57%)
12 (4.15%)
22 (7.61%)
1 (0.35%)
9 (3.11%)
4 (1.38%)
109
132
104
92
p=0.8882
NS
Shannon index of OTU level
p=0.8791
p=0.7372
p=3e-04
*
**
***
p=0.0001707
Chao index of OTU level
p=0.3578
Ace index of OTU level
R=0.0000, P=0.511000
PC1(38.4%)
PC2(25.81%)
R=0.3333, P=0.302000
PC1(40.32%)
PC2(20.13%)
R=0.5185, P=0.098000
PC1(45.59%)
PC2(18.8%)
R=0.8198, P=0.001000
PC1(24.25%)
PC2(13.12%)
stress :0.106, R=0.7811, P=0.001000
NMDS1
NMDS2
stress :0.051, R=0.8673, P=0.001000
