# Supplementary material for: Effects of Feeding Sources and Different Temperature Changes on the Gut Microbiome Structure of Chrysomya megacephala (Diptera: Calliphoridae)
Source: Insects. 2025 Mar 8;16(3):283. doi: 10.3390/insects16030283 (PMC11943086; doi:10.3390/insects16030283)
Supplement: Supplementary file 1 [file insects-16-00283-s001.zip › insects-3434865-supplementary/supplementary files/Figure S4.pdf]

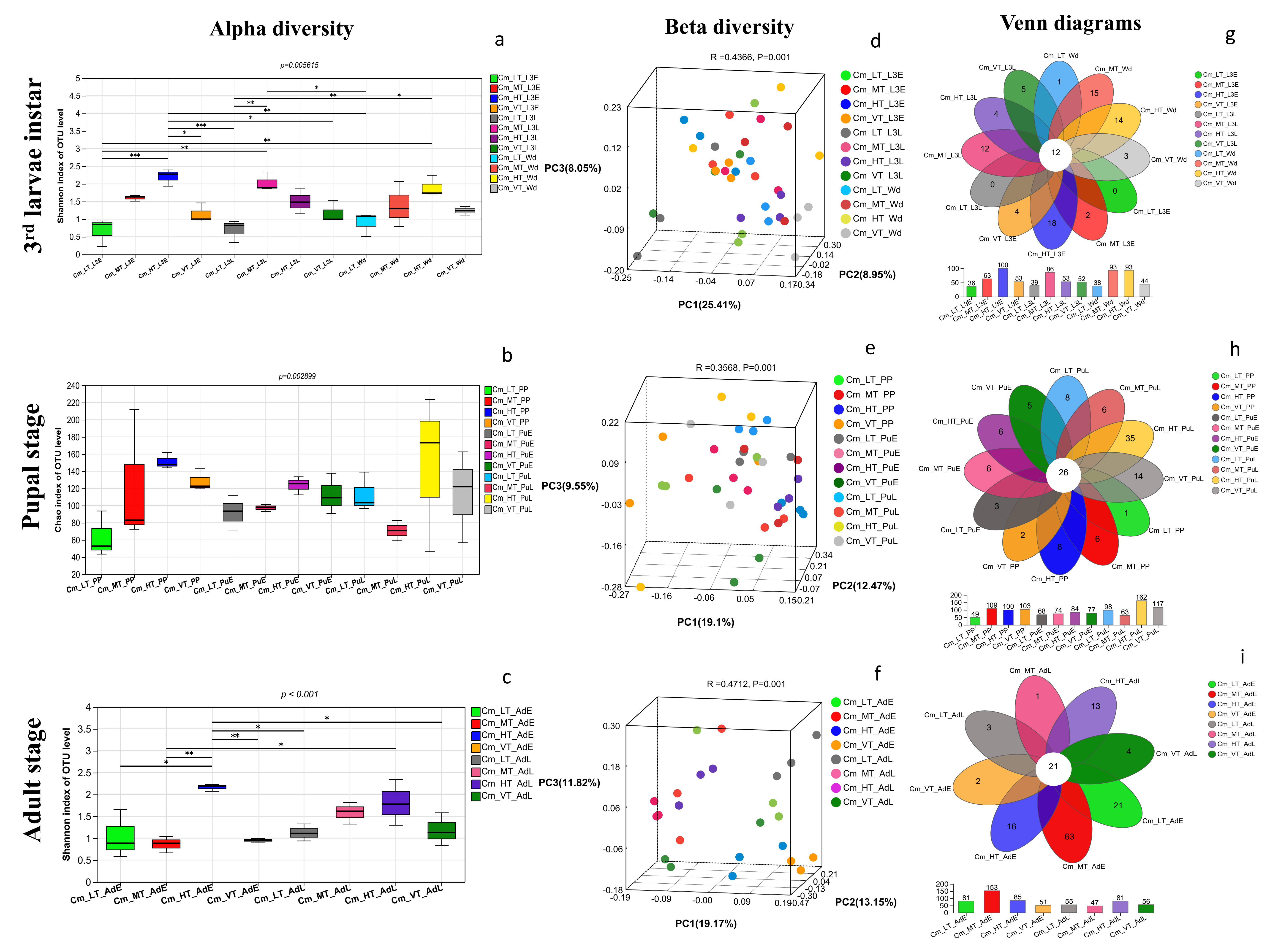
Alpha diversity
Beta diversity
Venn diagrams
3rd larvae instar
Pupal stage
Adult stage
a
b
c
d
e
f
g
h
i
p=0.005615
Shannon index of OTU level
Cm_LT_L3E
Cm_MT_L3E
Cm_HT_L3E
Cm_VT_L3E
Cm_LT_L3L
Cm_MT_L3L
Cm_HT_L3L
Cm_VT_L3L
Cm_LT_Wd
Cm_MT_Wd
Cm_HT_Wd
Cm_VT_Wd
p=0.002899
Chao index of OTU level
Cm_LT_PP
Cm_MT_PP
Cm_HT_PP
Cm_VT_PP
Cm_LT_PuE
Cm_MT_PuE
Cm_HT_PuE
Cm_VT_PuE
Cm_LT_PuL
Cm_MT_PuL
Cm_HT_PuL
Cm_VT_PuL
p < 0.001
Cm_LT_AdE
Cm_MT_AdE
Cm_HT_AdE
Cm_VT_AdE
Cm_LT_AdL
Cm_MT_AdL
Cm_HT_AdL
Cm_VT_AdL
R =0.4366, P=0.001
PC1(25.41%)
PC2(8.95%)
PC3(8.05%)
R =0.3568, P=0.001
PC1(19.1%)
PC2(12.47%)
PC3(9.55%)
R =0.4712, P=0.001
PC1(19.17%)
PC2(13.15%)
PC3(11.82%)
36 63 100 53 39 86 53 52 38 93 93 44
49 109 100 103 68 74 84 77 98 63 162 117
81 153 85 51 55 47 81 56
