# Supplementary material for: Effects of Feeding Sources and Different Temperature Changes on the Gut Microbiome Structure of Chrysomya megacephala (Diptera: Calliphoridae)
Source: Insects. 2025 Mar 8;16(3):283. doi: 10.3390/insects16030283 (PMC11943086; doi:10.3390/insects16030283)
Supplement: Supplementary file 1 [file insects-16-00283-s001.zip › insects-3434865-supplementary/supplementary files/Table S1-S16.pdf]

| Feeding sources | Development stages | Sample ID | OTU Number | Clean Sequences | Valid Sequences | Base number | Mean length | Min length | Max length |
|---|---|---|---|---|---|---|---|---|---|
| **Wheat bran and Fish meal** | Eggs | CMFEs_1 | 89 | 40646 | 29095 | 16468253 | 405.16 | 262 | 431 |
| | | CMFEs_2 | 201 | 45919 | 29095 | 18624369 | 405.59 | 366 | 451 |
| | | CMFEs_3 | 79 | 46880 | 29095 | 19143680 | 408.35 | 262 | 431 |
| | 1st instar Larvae | CMF1L_1 | 109 | 43390 | 29095 | 18550612 | 427.53 | 201 | 439 |
| | | CMF1L_2 | 93 | 43421 | 29095 | 18602857 | 428.43 | 232 | 439 |
| | | CMF1L_3 | 143 | 47525 | 29095 | 20372096 | 428.66 | 271 | 496 |
| | 2nd instar Larvae | CMF2L_1 | 88 | 41627 | 29095 | 17880951 | 429.55 | 314 | 436 |
| | | CMF2L_2 | 94 | 40804 | 29095 | 17522076 | 429.42 | 216 | 434 |
| | | CMF2L_3 | 101 | 44618 | 29095 | 19151404 | 429.23 | 230 | 434 |
| | 3rd instar Larvae Early | CMF3E_1 | 139 | 61068 | 29095 | 26044612 | 426.49 | 337 | 528 |
| | | CMF3E_2 | 119 | 91731 | 29095 | 39077418 | 426.00 | 201 | 432 |
| | | CMF3E_3 | 126 | 93419 | 29095 | 39951172 | 427.66 | 237 | 439 |
| | 3rd instar Larvae Late | CMF3L_1 | 96 | 57569 | 29095 | 24399214 | 423.83 | 317 | 440 |
| | | CMF3L_2 | 99 | 54074 | 29095 | 22699937 | 419.79 | 205 | 470 |
| | | CMF3L_3 | 106 | 69538 | 29095 | 29246369 | 420.58 | 259 | 434 |
| | Wandering | CMFW_1 | 197 | 53921 | 29095 | 22919118 | 425.05 | 286 | 439 |
| | | CMFW_2 | 225 | 42698 | 29095 | 18140998 | 424.87 | 212 | 439 |
| | | CMFW_3 | 204 | 44907 | 29095 | 19012810 | 423.38 | 225 | 432 |
| | Prepupae | CMFPP_1 | 86 | 57660 | 29095 | 24423758 | 423.58 | 337 | 441 |
| | | CMFPP_2 | 94 | 49186 | 29095 | 20957741 | 426.09 | 201 | 435 |
| | | CMFPP_3 | 98 | 59081 | 29095 | 25139649 | 425.51 | 265 | 439 |
| | Pupae Early | CMFPE_1 | 115 | 47128 | 29095 | 20074676 | 425.96 | 225 | 434 |

| | | | | | | | | | |
|---|---|---|---|---|---|---|---|---|---|
| | | CMFPE_2 | 102 | 129501 | 29095 | 55101546 | 425.49 | 251 | 449 |
| | | CMFPE_3 | 96 | 105400 | 29095 | 45028492 | 427.22 | 284 | 439 |
| | Pupae Late | CMFPL_1 | 35 | 40241 | 29095 | 17256900 | 428.84 | 262 | 436 |
| | | CMFPL_2 | 54 | 40316 | 29095 | 17286357 | 428.77 | 225 | 439 |
| | | CMFPL_3 | 51 | 40405 | 29095 | 17322929 | 428.73 | 370 | 432 |
| | Adult Early | CMFAE_1 | 90 | 51061 | 29095 | 21769659 | 426.35 | 202 | 440 |
| | | CMFAE_2 | 63 | 47451 | 29095 | 20306840 | 427.95 | 235 | 439 |
| | | CMFAE_3 | 60 | 45020 | 29095 | 19303319 | 428.77 | 262 | 430 |
| | Adult Late | CMFAL_1 | 73 | 53835 | 29095 | 23093088 | 428.96 | 225 | 437 |
| | | CMFAL_2 | 65 | 56310 | 29095 | 24139895 | 428.70 | 255 | 435 |
| | | CMFAL_3 | 52 | 53265 | 29095 | 22841418 | 428.83 | 251 | 480 |
| **Pork lungs** | Eggs | MB1_1 | 84 | 56337 | 29095 | 22882204 | 406.17 | 401 | 431 |
| | | MB1_2 | 58 | 51065 | 29095 | 20753064 | 406.40 | 330 | 431 |
| | | MB1_3 | 156 | 49013 | 29095 | 19979355 | 407.63 | 366 | 432 |
| | 1st instar Larvae | MB2_1 | 86 | 49084 | 29095 | 21068033 | 429.22 | 401 | 440 |
| | | MB2_2 | 108 | 34737 | 29095 | 14893335 | 428.75 | 403 | 432 |
| | | MB2_3 | 106 | 34854 | 29095 | 14947217 | 428.85 | 261 | 517 |
| | 2nd instar Larvae | MB3_1 | 80 | 33148 | 29095 | 14236266 | 429.48 | 402 | 431 |
| | | MB3_2 | 82 | 37225 | 29095 | 15992033 | 429.60 | 402 | 497 |
| | | MB3_3 | 70 | 35526 | 29095 | 15257467 | 429.47 | 402 | 431 |
| | 3rd instar Larvae Early | MB4_1 | 70 | 48692 | 29095 | 20814269 | 427.47 | 385 | 489 |
| | | MB4_2 | 76 | 38472 | 29095 | 16408756 | 426.51 | 402 | 434 |
| | | MB4_3 | 79 | 36612 | 29095 | 15648938 | 427.43 | 402 | 499 |
| | 3rd instar Larvae Late | MB5_1 | 57 | 52169 | 29095 | 21479701 | 411.73 | 370 | 511 |
| | | MB5_2 | 96 | 57498 | 29095 | 23958611 | 416.69 | 400 | 505 |
| | | MB5_3 | 99 | 40516 | 29095 | 16884417 | 416.73 | 400 | 433 |

| | | | | | | | | |
|---|---|---|---|---|---|---|---|---|
| Wandering | MB6_1 | 93 | 33066 | 29095 | 14006422 | 423.59 | 401 | 431 |
| | MB6_2 | 93 | 49309 | 29095 | 21064661 | 427.20 | 400 | 431 |
| | MB6_3 | 51 | 37437 | 29095 | 15499783 | 414.02 | 399 | 431 |
| Prepupae | MB7_1 | 157 | 34969 | 29095 | 14846126 | 424.55 | 394 | 433 |
| | MB7_2 | 64 | 30602 | 29095 | 13143104 | 429.49 | 402 | 449 |
| | MB7_3 | 61 | 32934 | 29095 | 14077893 | 427.46 | 402 | 431 |
| Pupae Early | MB8_1 | 79 | 38482 | 29095 | 15912884 | 413.51 | 337 | 431 |
| | MB8_2 | 81 | 38864 | 29095 | 16138452 | 415.25 | 345 | 432 |
| | MB8_3 | 80 | 38678 | 29095 | 15849260 | 409.77 | 401 | 431 |
| Pupae Late | MB9_1 | 59 | 58667 | 29095 | 23870863 | 406.89 | 401 | 430 |
| | MB9_2 | 46 | 60658 | 29095 | 25362662 | 418.13 | 345 | 492 |
| | MB9_3 | 71 | 62587 | 29095 | 25330424 | 404.72 | 399 | 430 |
| Adult Early | MB10_1 | 167 | 55485 | 29095 | 22608432 | 407.47 | 262 | 430 |
| | MB10_2 | 62 | 62404 | 29095 | 25392181 | 406.90 | 400 | 431 |
| | MB10_3 | 72 | 52553 | 29095 | 22221797 | 422.85 | 390 | 430 |
| Adult Late | MB11_1 | 76 | 61150 | 29095 | 26181948 | 428.16 | 262 | 469 |
| | MB11_2 | 74 | 67910 | 29095 | 28997840 | 427.00 | 274 | 474 |
| | MB11_3 | 64 | 68274 | 29095 | 29127797 | 426.63 | 403 | 431 |

**Table S1**: Statistical sequencing information across the life history of *C. megacephala* at different feeding sources.

| Temperatures (°C) | Development stages | Sample ID | OTU Number | Clean Sequence | Valid Sequence | Base number | Mean length | Min length | Max length |
|---|---|---|---|---|---|---|---|---|---|
| **LT (15)** | 3rd Larvae Early | MA4_1 | 43 | 32877 | 29095 | 14101589 | 428.92 | 233 | 431 |
| | | MA4_2 | 22 | 43848 | 29095 | 18849021 | 429.87 | 220 | 505 |

| | | | | | | | | | |
|---|---|---|---|---|---|---|---|---|---|
| | | MA4_3 | 27 | 34139 | 29095 | 14672308 | 429.78 | 403 | 470 |
| | 3rd Larvae Late | MA5_1 | 42 | 68043 | 29095 | 29186623 | 428.94 | 401 | 433 |
| | | MA5_2 | 44 | 41110 | 29095 | 17658026 | 429.53 | 401 | 431 |
| | | MA5_3 | 37 | 35996 | 29095 | 15466969 | 429.69 | 402 | 431 |
| | Wandering | MA6_1 | 47 | 36371 | 29095 | 15602497 | 428.98 | 390 | 432 |
| | | MA6_2 | 45 | 30561 | 29095 | 13104222 | 428.79 | 402 | 431 |
| | | MA6_3 | 55 | 37454 | 29095 | 16057064 | 428.71 | 337 | 431 |
| | Prepupae | MA7_1 | 68 | 32573 | 29095 | 13967560 | 428.81 | 400 | 431 |
| | | MA7_2 | 46 | 34658 | 29095 | 14889899 | 429.62 | 262 | 490 |
| | | MA7_3 | 37 | 33468 | 29095 | 14378950 | 429.63 | 403 | 431 |
| | Pupae Early | MA8_1 | 59 | 62876 | 29095 | 25611052 | 407.33 | 401 | 430 |
| | | MA8_2 | 84 | 37213 | 29095 | 15613844 | 419.58 | 345 | 432 |
| | | MA8_3 | 82 | 37305 | 29095 | 15371093 | 412.04 | 343 | 431 |
| | Pupae Late | MA9_1 | 97 | 50759 | 29095 | 21366438 | 420.94 | 392 | 431 |
| | | MA9_2 | 81 | 33808 | 29095 | 13714214 | 405.65 | 366 | 430 |
| | | MA9_3 | 97 | 32591 | 29095 | 13467312 | 413.22 | 345 | 431 |
| | Adult Early | MA10_1 | 65 | 34327 | 29095 | 14615984 | 425.79 | 262 | 433 |
| | | MA10_2 | 52 | 37937 | 29095 | 15446553 | 407.16 | 299 | 431 |
| | | MA10_3 | 72 | 35033 | 29095 | 14350676 | 409.63 | 401 | 430 |
| | Adult Late | MA11_1 | 50 | 36849 | 29095 | 15784907 | 428.37 | 403 | 431 |
| | | MA11_2 | 83 | 42933 | 29095 | 18420500 | 429.05 | 404 | 444 |
| | | MA11_3 | 73 | 36721 | 29095 | 15758779 | 429.15 | 403 | 431 |
| | 3rd Larvae Early | MB4_1 | 70 | 48692 | 29095 | 20814269 | 427.47 | 385 | 489 |
| | | MB4_2 | 76 | 38472 | 29095 | 16408756 | 426.51 | 402 | 434 |
| **MT(25)** | | MB4_3 | 79 | 36612 | 29095 | 15648938 | 427.43 | 402 | 499 |
| | 3rd Larvae Late | MB5_1 | 57 | 52169 | 29095 | 21479701 | 411.73 | 370 | 511 |
| | | MB5_2 | 96 | 57498 | 29095 | 23958611 | 416.69 | 400 | 505 |

| | | | | | | | | | |
|---|---|---|---|---|---|---|---|---|---|
| | | MB5_3 | 99 | 40516 | 29095 | 16884417 | 416.73 | 400 | 433 |
| | Wandering | MB6_1 | 93 | 33066 | 29095 | 14006422 | 423.59 | 401 | 431 |
| | | MB6_2 | 93 | 49309 | 29095 | 21064661 | 427.20 | 400 | 431 |
| | | MB6_3 | 51 | 37437 | 29095 | 15499783 | 414.02 | 399 | 431 |
| | Prepupae | MB7_1 | 157 | 34969 | 29095 | 14846126 | 424.55 | 394 | 433 |
| | | MB7_2 | 64 | 30602 | 29095 | 13143104 | 429.49 | 402 | 449 |
| | | MB7_3 | 61 | 32934 | 29095 | 14077893 | 427.46 | 402 | 431 |
| | Pupae Early | MB8_1 | 79 | 38482 | 29095 | 15912884 | 413.51 | 337 | 431 |
| | | MB8_2 | 81 | 38864 | 29095 | 16138452 | 415.25 | 345 | 432 |
| | | MB8_3 | 80 | 38678 | 29095 | 15849260 | 409.77 | 401 | 431 |
| | Pupae Late | MB9_1 | 59 | 58667 | 29095 | 23870863 | 406.89 | 401 | 430 |
| | | MB9_2 | 46 | 60658 | 29095 | 25362662 | 418.13 | 345 | 492 |
| | | MB9_3 | 71 | 62587 | 29095 | 25330424 | 404.72 | 399 | 430 |
| | Adult Early | MB10_1 | 167 | 55485 | 29095 | 22608432 | 407.47 | 262 | 430 |
| | | MB10_2 | 62 | 62404 | 29095 | 25392181 | 406.90 | 400 | 431 |
| | | MB10_3 | 72 | 52553 | 29095 | 22221797 | 422.85 | 390 | 430 |
| | Adult Late | MB11_1 | 76 | 61150 | 29095 | 26181948 | 428.16 | 262 | 469 |
| | | MB11_2 | 74 | 67910 | 29095 | 28997840 | 427.00 | 274 | 474 |
| | | MB11_3 | 64 | 68274 | 29095 | 29127797 | 426.63 | 403 | 431 |
| **HT (32)** | 3rd Larvae Early | MC4_1 | 72 | 67468 | 29095 | 28418224 | 421.21 | 343 | 431 |
| | | MC4_2 | 110 | 57593 | 29095 | 24263518 | 421.29 | 400 | 490 |
| | | MC4_3 | 112 | 67034 | 29095 | 27952900 | 417.00 | 296 | 431 |
| | 3rd Larvae Late | MC5_1 | 52 | 55144 | 29095 | 23105270 | 419.00 | 337 | 527 |
| | | MC5_2 | 49 | 63722 | 29095 | 27243794 | 427.54 | 401 | 432 |
| | | MC5_3 | 65 | 63840 | 29095 | 27131493 | 424.99 | 385 | 431 |
| | Wandering | MC6_1 | 68 | 41897 | 29095 | 17343930 | 413.97 | 366 | 431 |
| | | MC6_2 | 102 | 60885 | 29095 | 25331376 | 416.05 | 400 | 478 |

| | | | | | | | | | |
|---|---|---|---|---|---|---|---|---|---|
| | | MC6_3 | 62 | 43420 | 29095 | 18004569 | 414.66 | 370 | 440 |
| | Prepupae | MC7_1 | 72 | 53656 | 29095 | 22953450 | 427.79 | 401 | 431 |
| | | MC7_2 | 101 | 50436 | 29095 | 21566153 | 427.59 | 345 | 504 |
| | | MC7_3 | 107 | 56009 | 29095 | 24047638 | 429.35 | 402 | 448 |
| | Pupae Early | MC8_1 | 96 | 50946 | 29095 | 21575560 | 423.50 | 229 | 505 |
| | | MC8_2 | 78 | 70092 | 29095 | 29106873 | 415.27 | 398 | 431 |
| | | MC8_3 | 102 | 51994 | 29095 | 22020355 | 423.52 | 345 | 497 |
| | Pupae Late | MC9_2 | 147 | 68784 | 29095 | 28634659 | 416.30 | 400 | 432 |
| | | MC9_3 | 134 | 49406 | 29095 | 21000562 | 425.06 | 392 | 464 |
| | Adult Early | MC10_1 | 75 | 53351 | 29095 | 22441992 | 420.65 | 321 | 435 |
| | | MC10_2 | 89 | 54185 | 29095 | 22769178 | 420.21 | 400 | 430 |
| | | MC10_3 | 84 | 66573 | 29095 | 27996510 | 420.54 | 337 | 430 |
| | Adult Late | MC11_1 | 73 | 64829 | 29095 | 27791337 | 428.69 | 385 | 516 |
| | | MC11_2 | 68 | 51306 | 29095 | 21999956 | 428.80 | 403 | 430 |
| | | MC11_3 | 108 | 62660 | 29095 | 26816784 | 427.97 | 370 | 517 |
| | 3rd Larvae Early | MD4_1 | 73 | 41124 | 29095 | 17602310 | 428.03 | 401 | 434 |
| | | MD4_2 | 55 | 68102 | 29095 | 29243231 | 429.40 | 402 | 431 |
| | | MD4_3 | 52 | 54484 | 29095 | 23390532 | 429.31 | 402 | 431 |
| | 3rd Larvae Late | MD5_1 | 58 | 36962 | 29095 | 15696741 | 424.67 | 400 | 479 |
| | | MD5_2 | 59 | 50175 | 29095 | 21525145 | 429.00 | 394 | 492 |
| **VT (23.31)** | | MD5_3 | 57 | 67577 | 29095 | 28998004 | 429.11 | 402 | 436 |
| | Wandering | MD6_1 | 54 | 58697 | 29095 | 25019296 | 426.24 | 370 | 431 |
| | | MD6_2 | 56 | 86625 | 29095 | 37069342 | 427.93 | 283 | 434 |
| | | MD6_3 | 64 | 64460 | 29095 | 27503849 | 426.68 | 391 | 490 |
| | Prepupae | MD7_1 | 106 | 63118 | 29095 | 26681963 | 422.73 | 345 | 479 |
| | | MD7_2 | 97 | 53830 | 29095 | 23099408 | 429.12 | 397 | 478 |
| | | MD7_3 | 88 | 60072 | 29095 | 25790890 | 429.33 | 345 | 478 |

| | | | | | | | | |
|---|---|---|---|---|---|---|---|---|
| Pupae Early | MD8_1 | 82 | 66941 | 29095 | 27193459 | 406.23 | 382 | 450 |
| | MD8_2 | 77 | 68849 | 29095 | 28870982 | 419.34 | 400 | 501 |
| | MD8_3 | 87 | 67800 | 29095 | 28270287 | 416.97 | 317 | 431 |
| Pupae Late | MD9_1 | 97 | 45647 | 29095 | 18520086 | 405.72 | 400 | 431 |
| | MD9_2 | 140 | 44731 | 29095 | 18508508 | 413.77 | 345 | 431 |
| | MD9_3 | 48 | 43173 | 29095 | 17832257 | 413.04 | 401 | 430 |
| Adult Early | MD10_1 | 50 | 51656 | 29095 | 21670396 | 419.51 | 401 | 430 |
| | MD10_2 | 52 | 44081 | 29095 | 18195734 | 412.78 | 371 | 432 |
| | MD10_3 | 37 | 46494 | 29095 | 19364820 | 416.50 | 387 | 430 |
| Adult Late | MD11_1 | 65 | 75607 | 29095 | 32424989 | 428.86 | 274 | 473 |
| | MD11_2 | 64 | 37897 | 29095 | 16257986 | 429.00 | 403 | 431 |
| | MD11_3 | 67 | 42001 | 29095 | 18018839 | 429.01 | 403 | 478 |

**Table S2**: Statistical sequencing information across the life history of *C. megacephala* at different temperatures.

| Phylum (%) | Class (%) | Order (%) | Family (%) | Genus (%) | Species (%) |
|---|---|---|---|---|---|
| Proteobacteria (71.58)<br>Firmicutes (23.68)<br>Bacteroidota (4.36)<br>Actinobacteriota (0.33)<br>Unclassified Bacteria (0.009)<br>Other phyla (0.02) | Gammaproteobacteria (52.26)<br>Alphaproteobacteria (19.32)<br>Bacilli (17.26)<br>Clostridia (6.41)<br>Bacteroidia (4.36)<br>Other classes (0.37) | Cardiobacteriales (35.88)<br>Rickettsiales (17.80)<br>Lactobacillales (15.13)<br>Enterobacterales (12.55)<br>Peptostreptococcales-Tissierellales (6.33)<br>Other orders (12.23) | Wohlfahrtiimonadaceae (35.88)<br>Anaplasmataceae (17.80)<br>Morganellaceae (10.09)<br>Lactobacillaceae (7.68)<br>Family XI (5.64)<br>Other families (22.89) | *Ignatzschineria* (30.01)<br>*Wolbachia* (17.80)<br>*Providencia* (8.94)<br>*Weissella* (6.06)<br>*Wohlfahrtiimonas* (5.46)<br>Other genera (31.71) | *Ignatzschineria indica* (23.67)<br>*Wolbachia* spp. (17.80)<br>*Providencia* spp. (8.94)<br>*Weissella viridescens* (5.78)<br>*Wohlfahrtiimonas chitiniclastica* (5.46)<br>Other species (33.33) |

**Table S3:** Top five bacteria identified at different taxonomic levels in the gut microbiome of *C. megacephala* across all experimental conditions.

| Feeding sources | Phylum (%) | Class (%) | Order (%) | Family (%) | Genus (%) | Species (%) |
|---|---|---|---|---|---|---|
| **Wheat bran and Fish meal** | Proteobacteria (95.55)<br>Firmicutes (3.05)<br>Bacteroidota (0.68)<br>Cyanobacteria (0.25)<br>Chloroflexi (0.14)<br>Other phyla (0.30) | Alphaproteobacteria (90.40)<br>Gammaproteobacteria (5.14)<br>Bacilli (3.04)<br>Bacteroidia (0.68)<br>Cyanobacteriia (0.22)<br>Other classes (0.47) | Rickettsiales (88.55)<br>Lactobacillales (2.61)<br>Burkholderiales (2.01)<br>Acetobacterales (1.79)<br>Enterobacterales (1.21)<br>Other orders (3.80) | Anaplasmataceae (88.54)<br>Lactobacillaceae (2.21)<br>Alcaligenaceae (1.97)<br>Acetobacteraceae (1.79)<br>Morganellaceae (1.10)<br>Other families (4.36) | *Wolbachia* (88.54)<br>*Achromobacter* (1.97)<br>*Acetobacter* (1.79)<br>*Providencia* (0.85)<br>*Companilactobacillus* (0.68)<br>Other genera (6.14) | *Wolbachia* spp. (88.54)<br>*Achromobacter* spp. (1.97)<br>*Acetobacter indonesiensis* (1.79)<br>*Providencia* spp. (0.85)<br>*Lactobacillus crustorum* (0.68)<br>Other species (6.14) |
| **Pork lungs** | Proteobacteria (98.28)<br>Firmicutes (0.72)<br>Actinobacteriota (0.52)<br>Bacteroidota (0.37)<br>Deferribacterota (0.01)<br>Other phyla (0.08) | Alphaproteobacteria (88.41)<br>Gammaproteobacteria (9.86)<br>Bacilli (0.52)<br>Actinobacteria (0.47)<br>Bacteroidia (0.37)<br>Other classes (0.34) | Rickettsiales (87.67)<br>Burkholderiales (5.92)<br>Pseudomonadales (2.81)<br>Cardiobacteriales (1.04)<br>Sphingomonadales (0.45)<br>Other orders (2.08) | Anaplasmataceae (87.67)<br>Alcaligenaceae (5.62)<br>Pseudomonadaceae (2.73)<br>Wohlfahrtiimonadaceae (1.04)<br>Sphingomonadaceae (0.45)<br>Other families (2.47) | *Wolbachia* (87.67)<br>*Achromobacter* (5.62)<br>*Pseudomonas* (2.73)<br>*Wohlfahrtiimonas* (0.88)<br>*Blastomonas* (0.40)<br>Other genera (2.67) | *Wolbachia* spp. (87.67)<br>*Achromobacter* spp. (5.62)<br>*Pseudomonas* spp. (2.59)<br>*Wohlfahrtiimonas chitiniclastica* (0.88)<br>*Blastomonas* spp. (0.40)<br>Other species (2.82) |

**Table S4:** Top five bacteria identified at different taxonomic levels in the eggs of *C. megacephala* collected on different feeding sources.

| Feeding sources | Phylum (%) | Class (%) | Order (%) | Family (%) | Genus (%) | Species (%) |
|---|---|---|---|---|---|---|
| **Wheat bran and Fish meal** | Proteobacteria (79.11)<br>Firmicutes (19.87)<br>Bacteroidota (0.98)<br>Actinobacteriota (0.009)<br>Unclassified Bacteria (0.006)<br>Other phyla (0.002) | Gammaproteobacteria (73.99)<br>Bacilli (19.86)<br>Alphaproteobacteria (5.11)<br>Bacteroidia (0.98)<br>Actinobacteria (0.009)<br>Other classes (0.01) | Cardiobacteriales (55.06)<br>Lactobacillales (19.43)<br>Enterobacterales (12.47)<br>Rickettsiales (4.69)<br>Pseudomonadales (4.25)<br>Other orders (4.06) | Wohlfahrtiimonadaceae (55.06)<br>Lactobacillaceae (18.35)<br>Enterobacteriaceae (8.77)<br>Anaplasmataceae (4.69)<br>Moraxellaceae (3.86)<br>Other families (9.24) | *Wohlfahrtiimonas* (52.69)<br>*Weissella* (17.62)<br>*Enterobacter* (8.02)<br>*Wolbachia* (4.69)<br>*Acinetobacter* (3.79)<br>Other genera (13.16) | *Wohlfahrtiimonas chitiniclastica* (52.69)<br>*Weissella viridescens* (17.27)<br>*Klebsiella quasipneumoniae* (7.00)<br>*Wolbachia* spp. (4.69)<br>*Acinetobacter* spp. (3.60)<br>Other species (14.71) |
| **Pork lungs** | Proteobacteria (71.62)<br>Firmicutes (22.82) | Gammaproteobacteria (71.39) | Cardiobacteriales (48.92) | Wohlfahrtiimonadaceae (48.92) | *Wohlfahrtiimonas* (46.26) | *Wohlfahrtiimonas chitiniclastica* (46.26) |

| | | | | | | |
|---|---|---|---|---|---|---|
| | Bacteroidota (4.65)<br>Actinobacteriota (0.87)<br>Acidobacteriota (0.01)<br>Other phyla (0.006) | Bacilli (22.10)<br>Bacteroidia (4.65)<br>Actinobacteria (0.87)<br>Clostridia (0.71)<br>Other classes (0.24) | Pseudomonadales (20.31)<br>Lactobacillales (13.08)<br>Staphylococcales (7.20)<br>Flavobacteriales (4.46)<br>Other orders (6.00) | Moraxellaceae (19.98)<br>Staphylococcaceae (7.20)<br>Lactobacillaceae (6.83)<br>Flavobacteriaceae (4.31)<br>Other families (12.73) | *Acinetobacter* (15.31)<br>*Macrococcus* (7.19)<br>*Weissella* (6.83)<br>*Psychrobacter* (4.47)<br>Other genera 19.91) | *Acinetobacter* spp. (12.54)<br>*Macrococcus caseolyticus* (7.19)<br>*Weissella ceti* (6.02)<br>*Psychrobacter* spp. (4.47)<br>Other species (23.49) |

**Table S5:** Top five bacteria identified at different taxonomic levels during the first instar larvae (L1) of *C. megacephala* collected on different feeding sources.

| Feeding sources | Phylum (%) | Class (%) | Order (%) | Family (%) | Genus (%) | Species (%) |
|---|---|---|---|---|---|---|
| **Wheat bran and Fish meal** | Proteobacteria (72.31)<br>Firmicutes 26.80)<br>Bacteroidota (0.86)<br>Unclassified Bacteria (0.01) | Gammaproteobacteria (71.88)<br>Bacilli (26.80)<br>Bacteroidia (0.86)<br>Alphaproteobacteria (0.42)<br>Unclassified Bacteria (0.01)<br>Other classes (0.02) | Cardiobacteriales (63.40)<br>Lactobacillales (26.79)<br>Enterobacterales (8.05)<br>Flavobacteriales (0.86)<br>Rickettsiales (0.31)<br>Other orders (0.56) | Wohlfahrtiimonadaceae (63.40)<br>Lactobacillaceae (24.78)<br>Morganellaceae (6.90)<br>Vagococcaceae (1.24)<br>Enterobacteriaceae (0.85)<br>Other families (2.79) | *Ignatzschineria* (39.86)<br>*Wohlfahrtiimonas* (22.30)<br>*Weissella* (21.02)<br>*Providencia* (6.46)<br>*Pediococcus* (1.57)<br>Other genera (8.77) | *Wohlfahrtiimonas chitiniclastica* (22.30)<br>*Ignatzschineria ureiclastica* (21.36)<br>*Weissella viridescens* (19.56)<br>*Ignatzschineria indica* (15.28)<br>*Providencia* spp. (6.44)<br>Other species (14.94) |
| **Pork lungs** | Proteobacteria (92.49)<br>Firmicutes (6.27) | Gammaproteobacteria (9248) | Cardiobacteriales (92.17) | Wohlfahrtiimonadaceae (92.17) | *Wohlfahrtiimonas* (62.50) | *Wohlfahrtiimonas chitiniclastica* (62.50) |

| | | | | | | | |
|---|---|---|---|---|---|---|---|
| | | Bacteroidota (1.12)<br>Actinobacteriota (0.1) | Bacilli (5.26)<br>Bacteroidia (1.12)<br>Actinobacteria (0.1)<br>Alphaproteobacteria (0.01) | Lactobacillales (5.07)<br>Flavobacteriales (1.07)<br>Peptostreptococcales-Tissierellales (0.90)<br>Erysipelotrichales (0.16)<br>Other orders (0.60) | Vagococcaceae (4.56)<br>Flavobacteriaceae (1.07)<br>Family_XI (0.70)<br>Streptococcaceae (0.38)<br>Other families (1.09) | *Ignatzschineria* (29.64)<br>*Vagococcus* (4.56)<br>*Myroides* (1.07)<br>*Peptoniphilus* (0.32)<br>Other genera (1.89) | *Ignatzschineria indica* (16.04)<br>*Ignatzschineria ureiclastica* (7.74)<br>*Ignatzschineria* spp. (5.84)<br>*Vagococcus* spp. (4.51)<br>Other species (3.33) |

**Table S6:** Top five bacteria identified at different taxonomic levels during the second instar larvae (L2) of *C. megacephala* collected on different feeding sources.

| Feeding sources | Sub development-stages | Phylum (%) | Class (%) | Order (%) | Family (%) | Genus (%) | Species (%) |
|---|---|---|---|---|---|---|---|
| **Wheat bran and Fish meal** | Third larvae early (L3E) | Proteobacteria (67.12)<br>Firmicutes (32.57)<br>Bacteroidota (0.21)<br>Unclassified Bacteria (0.08) | Gammaproteobacteria (57.11)<br>Bacilli (32.57)<br>Alphaproteobacteria (9.99)<br>Bacteroidia (0.21)<br>Unclassified Bacteria (0.08)<br>Other classes (0.009) | Lactobacillales (32.56)<br>Cardiobacteriales (28.48)<br>Enterobacterales (27.40)<br>Acetobacterales (9.99)<br>Unclassified Gammaproteobacteria (1.20) | Lactobacillaceae (32.19)<br>Wohlfahrtiimonadaceae (28.48)<br>Morganellaceae (23.83)<br>Acetobacteraceae (9.99)<br>Enterobacteriaceae (2.80)<br>Other families (2.68) | *Providencia* (19.33)<br>*Wohlfahrtiimonas* (15.61)<br>*Weissella* (13.72)<br>*Ignatzschineria* (11.70)<br>*Acetobacter* (9.98)<br>Other genera (29.64) | *Providencia* spp. (19.30)<br>*Wohlfahrtiimonas chitiniclastica* (15.61)<br>*Weissella viridescens* (12.97)<br>*Acetobacter indonesiensis* (9.79)<br>*Lactobacillus brevis* |

| | | | | | | | |
|---|---|---|---|---|---|---|---|
| | | | | Other orders (0.34) | | | (7.03)<br>Other species (35.27) |
| | Third larvae late (L3L) | Proteobacteria (64.71)<br>Firmicutes (24.67)<br>Bacteroidota (10.54)<br>Unclassified Bacteria (0.06)<br>Actinobacteriota (0.001) | Gammaproteobacteria (33.90)<br>Alphaproteobacteria (30.80)<br>Bacilli (24.67)<br>Bacteroidia (10.54)<br>Unclassified Bacteria (0.06)<br>Other classes (0.01) | Acetobacterales (30.80)<br>Lactobacillales (24.64)<br>Cardiobacteriales (17.65)<br>Enterobacterales (15.71)<br>Bacteroidales (10.52)<br>Other orders (0.62) | Acetobacteraceae (30.80)<br>Lactobacillaceae (23.17)<br>Wohlfahrtiimonadaceae (17.65)<br>Morganellaceae (15.07)<br>Dysgonomonadaceae (10.52)<br>Other families (2.75) | *Acetobacter* (30.79)<br>*Providencia* (12.45)<br>*Dysgonomonas* (10.52)<br>*Wohlfahrtiimonas* (10.36)<br>*Levilactobacillus* (7.49)<br>Other genera (28.35) | *Acetobacter indonesiensis* (30.59)<br>*Providencia* spp. (12.44)<br>*Dysgonomonas* spp. (10.52)<br>*Wohlfahrtiimonas chitiniclastica* (10.36)<br>*Lactobacillus brevis* (7.34)<br>Other species (28.61) |
| | Wandering (Wd) | Proteobacteria (50.69)<br>Bacteroidota (24.90)<br>Firmicutes (23.99)<br>Actinobacteriota (0.28)<br>Unclassified Bacteria (0.12)<br>Other Phyla (0.002) | Gammaproteobacteria (36.12)<br>Bacteroidia (24.90)<br>Bacilli (23.84)<br>Alphaproteobacteria (14.55)<br>Actinobacteria (0.28)<br>Other classes (0.28) | Bacteroidales (22.66)<br>Acetobacterales (14.20)<br>Enterobacterales (21.60)<br>Lactobacillales (23.82)<br>Cardiobacteriales (9.57)<br>Other orders (8.12) | Dysgonomonadaceae (22.48)<br>Lactobacillaceae (22.01)<br>Morganellaceae (15.73)<br>Acetobacteraceae (14.20)<br>Wohlfahrtiimonadaceae (9.57)<br>Other families (15.98) | *Dysgonomonas* (22.47)<br>*Acetobacter* (14.15)<br>*Providencia* (12.91)<br>*Levilactobacillus* (8.16)<br>*Wohlfahrtiimonas* (6.09)<br>Other genera (36.19) | *Dysgonomonas* spp. (16.28)<br>*Acetobacter indonesiensis* (13.87)<br>*Providencia* spp. (12.90)<br>*Lactobacillus brevis* (8.12)<br>*Dysgonomonas capnocytophagoides* (6.19)<br>Other species (42.62) |
| | Third larvae early (L3E) | Proteobacteria (73.64)<br>Firmicutes (26.31)<br>Actinobacteriota (0.02)<br>Bacteroidota (0.008) | Gammaproteobacteria (73.62)<br>Bacilli (17.49)<br>Clostridia (8.82)<br>Actinobacteria (0.029)<br>Alphaproteobacteria (0.025)<br>Other Classes (0.008) | Cardiobacteriales (73.54)<br>Lactobacillales (12.61)<br>Peptostreptococcales-Tissierellales (8.79)<br>Flavobacteriales (1.07)<br>Staphylococcales (3.56)<br>Bacillales (0.80)<br>Other orders (0.67) | Wohlfahrtiimonadaceae (73.54)<br>Vagococcaceae (12.17)<br>Family_XI (8.55)<br>Staphylococcaceae (3.56)<br>Planococcaceae (0.79)<br>Other families (1.36) | *Ignatzschineria* (73.51)<br>*Vagococcus* (12.17)<br>*Gallicola* (5.14)<br>*Staphylococcus* (3.53)<br>*Tissierella* (1.61)<br>Other genera (4.02) | *Ignatzschineria indica* (40.01)<br>*Ignatzschineria ureiclastica* (33.47)<br>*Vagococcus* spp. (12.07)<br>*Gallicola* spp. (5.14)<br>*Staphylococcus nepalensis* (1.94)<br>Other species (7.34) |
| **Pork lungs** | Third larvae late (L3L) | Firmicutes (65.27)<br>Proteobacteria (34.66)<br>Actinobacteriota (0.05)<br>Bacteroidota (0.01)<br>Cyanobacteria (0.001) | Clostridia (54.98)<br>Gammaproteobacteria (34.63)<br>Bacilli (10.28)<br>Actinobacteria (0.04)<br>Alphaproteobacteria (0.02)<br>Other Classes (0.02) | Peptostreptococcales-Tissierellales 54.94)<br>Cardiobacteriales (34.41)<br>Lactobacillales (8.07)<br>Erysipelotrichales (1.12)<br>Staphylococcales | Family_XI (52.05)<br>Wohlfahrtiimonadaceae (34.41)<br>Vagococcaceae (5.83)<br>Peptostreptococcaceae (2.88)<br>Lactobacillaceae (1.70)<br>Other families (3.10) | *Ignatzschineria* (34.40)<br>*Gallicola* (30.10)<br>Unclassified *Family_XI* (11.45)<br>*Tissierella* (9.85)<br>*Vagococcus* (5.83)<br>Other genera (8.34) | *Gallicola* spp. (30.10)<br>*Ignatzschineria indica* (21.11)<br>*Ignatzschineria ureiclastica* (13.28)<br>*Family_XI* spp. (11.45)<br>*Tissierella* spp. (9.67) |

| | | | | | | | |
|---|---|---|---|---|---|---|---|
| | | | | (0.97)<br>Other orders (0.47) | | | Other species (14.36) |
| | Wandering (Wd) | Proteobacteria (63.68)<br>Firmicutes (36.10)<br>Actinobacteriota (0.18)<br>Bacteroidota (0.02)<br>Chloroflexi (0.003)<br>Other Phyla (0.003) | Gammaproteobacteria (63.67)<br>Clostridia (30.65)<br>Bacilli (5.45)<br>Actinobacteria (0.18)<br>Bacteroidia (0.02)<br>Other Classes (0.01) | Cardiobacteriales (63.56)<br>Peptostreptococcales-Tissierellales (30.60)<br>Lactobacillales (4.51)<br>Erysipelotrichales (0.54)<br>Bacillales (0.35)<br>Other orders (0.41) | Wohlfahrtiimonadaceae (63.56)<br>Family_XI (25.92)<br>Peptostreptococcaceae (4.67)<br>Vagococcaceae (4.40)<br>Erysipelotrichaceae (0.51)<br>Other families (0.90) | *Ignatzschineria* (63.56)<br>Unclassified *Family_XI* (14.30)<br>*Gallicola* (5.90)<br>*Tissierella* (4.83)<br>*Peptostreptococcus* (4.53)<br>Other genera (6.85) | *Ignatzschineria indica* (57.69)<br>*Family_XI* spp. (14.30)<br>*Gallicola* spp. (5.90)<br>*Ignatzschineria ureiclastica* (5.84)<br>*Tissierella* spp. (4.70)<br>Other species (11.54) |

**Table S7:** Top five bacteria identified at different taxonomic levels during the third instar larvae (L3) of *C. megacephala* collected on different feeding sources.

| Feeding sources | Sub development-stages | Phylum (%) | Class (%) | Order (%) | Family (%) | Genus (%) | Species (%) |
|---|---|---|---|---|---|---|---|
| **Wheat bran and Fish meal** | Prepupae (PP) | Proteobacteria (92.01)<br>Bacteroidota (6.68)<br>Firmicutes (1.27)<br>Unclassified Bacteria (0.02)<br>Actinobacteriota (0.009) | Gammaproteobacteria (75.90)<br>Alphaproteobacteria (16.01)<br>Bacteroidia (6.68)<br>Bacilli (1.26)<br>Unclassified Bacteria (0.02)<br>Other classes (0.01) | Enterobacterales (53.94)<br>Cardiobacteriales (18.90)<br>Rickettsiales (15.97)<br>Bacteroidales (6.21)<br>Pseudomonadales (2.42)<br>Other orders (2.52) | Morganellaceae (38.94)<br>Wohlfahrtiimonadaceae (18.90)<br>Anaplasmataceae (15.97)<br>Shewanellaceae (13.43)<br>Dysgonomonadaceae (6.20)<br>Other families (6.53) | *Providencia* (38.83)<br>*Wolbachia* (15.97)<br>*Shewanella* (13.43)<br>*Wohlfahrtiimonas* (11.81)<br>*Dysgonomonas* (6.20)<br>Other genera (13.73) | *Providencia* spp. (38.83)<br>*Wolbachia* spp. (15.97)<br>*Shewanella* sp. WE21 (12.49)<br>*Wohlfahrtiimonas chitiniclastica* (11.81)<br>*Dysgonomonas* spp. (6.18)<br>Other species (14.69) |
| | Pupae Early (PuE) | Proteobacteria (94.12) | Gammaproteobacteria (83.31) | Enterobacterales (69.99) | Morganellaceae (69.00)<br>Wohlfahrtiimonadaceae | *Providencia* (58.79)<br>*Wolbachia* (10.71) | *Providencia* spp. (58.78) |

| | | | | | | | |
|---|---|---|---|---|---|---|---|
| | | Firmicutes (3.91)<br>Bacteroidota (2.66)<br>Unclassified Bacteria (0.02)<br>Actinobacteriota (0.002) | Alphaproteobacteria (10.80)<br>Bacilli (3.19)<br>Bacteroidia (2.66)<br>Unclassified Bacteria (0.02)<br>Other classes (0.002) | Cardiobacteriales (10.92)<br>Rickettsiales (10.71)<br>Lactobacillales (3.17)<br>Bacteroidales (1.71)<br>Other orders (3.48) | (10.92)<br>Anaplasmataceae (10.71)<br>Vagococcaceae (2.71)<br>Dysgonomonadaceae (1.71)<br>Other families (4.84) | *Morganella* (10.02)<br>*Wohlfahrtiimonas* (7.37)<br>*Koukoulia* (2.98)<br>Other genera (10.09) | *Wolbachia* spp. (10.71)<br>*Morganella* spp. (10.02)<br>*Wohlfahrtiimonas chitiniclastica* (7.37)<br>*Koukoulia aurantiaca* (2.98)<br>Other species (10.11) |
| | Pupae Late (PuL) | Proteobacteria (90.35)<br>Firmicutes (9.47)<br>Bacteroidota (0.14)<br>Unclassified Bacteria (0.02)<br>Actinobacteriota (0.001) | Gammaproteobacteria (89.71)<br>Bacilli (9.47)<br>Alphaproteobacteria (0.63)<br>Bacteroidia (0.14)<br>Unclassified Bacteria (0.02)<br>Other classes (0.001) | Enterobacterales (88.64)<br>Lactobacillales (9.44)<br>Cardiobacteriales (0.82)<br>Rickettsiales (0.63)<br>Unclassified Gammaproteobacteria (0.15)<br>Other orders (0.29) | Morganellaceae (87.87)<br>Vagococcaceae (8.98)<br>Wohlfahrtiimonadaceae (0.82)<br>Anaplasmataceae (0.63)<br>Enterococcaceae (046)<br>Other families (1.22) | *Providencia* (77.62)<br>*Morganella* (10.20)<br>*Vagococcus* (8.98)<br>*Wolbachia* (0.63)<br>*Enterococcus* (0.46)<br>Other genera (2.08) | *Providencia* spp. (77.61)<br>*Morganella* spp. (10.20)<br>*Vagococcus fluvialis* (7.97)<br>*Vagococcus* spp. (0.97)<br>*Wolbachia* spp. (0.63)<br>Other species (2.59) |
| | Prepupae (PP) | Proteobacteria (77.88)<br>Firmicutes (20.37)<br>Bacteroidota (1.49)<br>Actinobacteriota (0.23)<br>Acidobacteriota (0.004)<br>Other Phyla (0.004) | Gammaproteobacteria (77.74)<br>Bacilli (11.32)<br>Clostridia (9.02)<br>Bacteroidia (1.49)<br>Actinobacteria (0.23)<br>Other classes (0.16) | Cardiobacteriales (71.07)<br>Peptostreptococcales-Tissierellales (9.01)<br>Lactobacillales (7.84)<br>Enterobacterales (6.49)<br>Bacillales (3.11)<br>Other orders (2.45) | Wohlfahrtiimonadaceae (71.07)<br>Vagococcaceae (7.20)<br>Morganellaceae (6.46)<br>Family_XI (5.43)<br>Peptostreptococcaceae (3.57)<br>Other families (6.24) | *Ignatzschineria* (70.23)<br>*Vagococcus* (7.20)<br>*Providencia* (5.02)<br>Unclassified *Family_XI* (3.71)<br>*Peptostreptococcus* (3.56)<br>Other genera (10.25) | *Ignatzschineria indica* (63.06)<br>*Vagococcus* spp. (6.72)<br>*Ignatzschineria ureiclastica* (6.57)<br>*Providencia* spp. (5.02)<br>*Family_XI* spp. (3.71)<br>Other species (14.89) |
| **Pork lungs** | Pupae Early (PuE) | Proteobacteria (86.36)<br>Firmicutes (10.61)<br>Bacteroidota (2.78)<br>Actinobacteriota (1.18)<br>Fusobacteriota (0.04)<br>Other Phyla (0.006) | Alphaproteobacteria (60.69)<br>Gammaproteobacteria (25.67)<br>Bacilli (5.95)<br>Clostridia (4.66)<br>Bacteroidia (2.78)<br>Other Classes (0.23) | Rickettsiales (60.59)<br>Cardiobacteriales (16.50)<br>Enterobacterales (7.84)<br>Peptostreptococcales-Tissierellales (4.65)<br>Lactobacillales (8.07)<br>Bacillales (3.50)<br>Other orders (6.89) | Anaplasmataceae (60.59)<br>Wohlfahrtiimonadaceae (16.50)<br>Morganellaceae (7.64)<br>Family_XI (4.64)<br>Planococcaceae (3.50)<br>Other families (7.11) | *Wolbachia* (60.59)<br>*Ignatzschineria* (16.01)<br>*Providencia* (6.91)<br>Unclassified *Family_XI* (3.57)<br>*Sporosarcina* (3.06)<br>Other genera (9.83) | *Wolbachia* spp. (60.59)<br>*Ignatzschineria indica* (8.41)<br>*Swine effluent bacterium CHNDP41* (7.54)<br>*Providencia* spp. (6.91)<br>*Family_XI* spp. (3.57)<br>Other species (12.95) |
| | Pupae Late | Proteobacteria | Alphaproteobacteria | Rickettsiales (64.62) | Anaplasmataceae (64.62) | *Wolbachia* (64.62) | *Wolbachia* spp. |

| | | | | | | | |
|---|---|---|---|---|---|---|---|
| | (PuL) | (66.70)<br>Firmicutes (32.85)<br>Bacteroidota 0.32)<br>Actinobacteriota (0.11) | (64.80)<br>Bacilli (21.62)<br>Clostridia (11.23)<br>Gammaproteobacteria (1.89)<br>Bacteroidia (0.32)<br>Other Classes (0.01) | Lactobacillales (21.56)<br>Peptostreptococcales-Tissierellales (11.23)<br>Enterobacterales (1.20)<br>Pseudomonadales (0.46)<br>Other orders (0.90) | Vagococcaceae (21.54)<br>Peptostreptococcaceae (10.66)<br>Morganellaceae (1.05)<br>Family_XI (0.57)<br>Other families (1.54) | Vagococcus (21.54)<br>*Paraclostridium* (10.52)<br>*Providencia* (0.73)<br>*Pseudomonas* (0.37)<br>Other genera (2.19) | (64.62)<br>*Vagococcus* spp. (17.37)<br>*Paraclostridium bifermentans*. (10.50)<br>*Vagococcus fluvialis* (4.00)<br>*Providencia* spp. (0.73)<br>Other species (2.75) |

**Table S8:** Top five bacteria identified at different taxonomic levels during the pupal stages of *C. megacephala* collected on different feeding sources.

| Feeding sources | Sub development-stages | Phylum (%) | Class (%) | Order (%) | Family (%) | Genus (%) | Species (%) |
|---|---|---|---|---|---|---|---|
| **Wheat bran and Fish meal** | Adult Early (AdE) | Proteobacteria (93.43)<br>Bacteroidota (2.66)<br>Firmicutes (0.26)<br>Actinobacteriota (0.009)<br>Unclassified Bacteria (0.001) | Gammaproteobacteria (89.11)<br>Bacteroidia (6.29)<br>Alphaproteobacteria (4.23)<br>Bacilli (0.26)<br>Actinobacteria (0.009)<br>Other classes (0.001) | Enterobacterales (77.77)<br>Pseudomonadales (8.10)<br>Bacteroidales (5.01)<br>Rickettsiales (4.27)<br>Cardiobacteriales (2.95)<br>Other orders (1.87) | Morganellaceae (59.85)<br>Shewanellaceae (16.34)<br>Pseudomonadaceae (7.53)<br>Dysgonomonadaceae (5.01)<br>Anaplasmataceae (4.27)<br>Other families (6.97) | *Providencia* (56.18)<br>*Shewanella* (16.34)<br>*Pseudomonas* (7.53)<br>*Dysgonomonas* (7.53)<br>*Wolbachia* (4.27)<br>Other genera (10.64) | *Providencia* spp. (56.18)<br>*Shewanella* sp. WE21 (11.90)<br>*Pseudomonas* spp. (7.50)<br>*Dysgonomonas* spp. (4.65)<br>*Wolbachia* spp. (4.27)<br>Other species (15.48) |
| | Adult Late (AdL) | Firmicutes (94.14)<br>Proteobacteria (5.37)<br>Bacteroidota (0.46)<br>Unclassified Bacteria (0.005)<br>Patescibacteria (0.003)<br>Other Phyla (0.003) | Bacilli (94.14)<br>Gammaproteobacteria (4.79)<br>Alphaproteobacteria (0.58)<br>Bacteroidia (0.46)<br>Unclassified Bacteria (0.005)<br>Other Classes (0.006) | Lactobacillales (94.14)<br>Enterobacterales (3.04)<br>Pseudomonadales (0.92)<br>Cardiobacteriales (0.79)<br>Rickettsiales (0.52)<br>Other orders (0.56) | Lactobacillaceae (70.23)<br>Vagococcaceae (15.97)<br>Enterococcaceae (7.29)<br>Morganellaceae (1.30)<br>Enterobacteriaceae (0.92)<br>Other families (4.26) | *Weissella* (68.57)<br>*Vagococcus* (15.97)<br>*Enterococcus* (7.29)<br>*Morganella* (1.06)<br>*Pseudomonas* (0.88)<br>Other genera (6.20) | *Weissella viridescens* (66.54)<br>*Vagococcus* spp. (13.25)<br>*Enterococcus sulfureus* (5.87)<br>Unclassified *Vagococcus* (2.71)<br>*Weissella* spp. (2.03)<br>Other species (9.58) |
| **Pork lungs** | Adult Early (AdE) | Proteobacteria (71.03) | Alphaproteobacteria (65.84) | Rickettsiales (64.81)<br>Staphylococcales | Anaplasmataceae (64.81)<br>Staphylococcaceae | *Wolbachia* (64.81)<br>*Staphylococcus* | *Wolbachia* spp. (64.81) |

| | | | | | | |
|---|---|---|---|---|---|---|
| | Firmicutes (27.33)<br>Bacteroidota (0.93)<br>Actinobacteriota (0.62)<br>Deinococcota (0.01)<br>Other Phyla (0.06) | Bacilli (27.25)<br>Gammaproteobacteria (5.19)<br>Bacteroidia (0.92)<br>Actinobacteria (0.61)<br>Other Classes (0.17) | (26.44)<br>Burkholderiales (3.35)<br>Pseudomonadales (1.30)<br>Flavobacteriales (0.87)<br>Other orders (3.20) | (26.44)<br>Alcaligenaceae (2.87)<br>Pseudomonadaceae (0.99)<br>Weeksellaceae (0.85)<br>Other families (4.01) | (26.36)<br>*Achromobacter* (2.87)<br>*Pseudomonas* (0.99)<br>*Chryseobacterium* (0.81)<br>Other genera (4.13) | *Staphylococcus nepalensis* (23.27)<br>*Staphylococcus xylosus* (2.96)<br>*Achromobacter* spp. (2.87)<br>*Chryseobacterium indologenes* (0.81)<br>Other species (5.26) |
| Adult Late (AdL) | Firmicutes (54.09)<br>Proteobacteria (45.01)<br>Bacteroidota (0.84)<br>Actinobacteriota (0.05) | Bacilli (54.08)<br>Gammaproteobacteria (43.27)<br>Alphaproteobacteria (1.73)<br>Bacteroidia (0.84)<br>Actinobacteria (0.05)<br>Other Classes (0.004) | Lactobacillales (53.25)<br>Pseudomonadales (39.77)<br>Cardiobacteriales (2.73)<br>Rickettsiales (1.60)<br>Staphylococcales (0.83)<br>Other orders (1.80) | Pseudomonadaceae (39.46)<br>Enterococcaceae (30.45)<br>Vagococcaceae (14.48)<br>Lactobacillaceae (6.33)<br>Wohlfahrtiimonadaceae (2.73)<br>Other families (6.52) | *Pseudomonas* (39.46)<br>*Enterococcus* (30.45)<br>*Vagococcus* (14.48)<br>*Weissella* (5.25)<br>*Ignatzschineria* (2.73)<br>Other genera (7.60) | *Gammaproteobacteria bacterium* ESL0073 (39.26)<br>*Enterococcus sulfureus* (30.36)<br>*Vagococcus* spp. (14.19)<br>*Weissella viridescens* (5.17)<br>*Ignatzschineria indica* (2.26)<br>Other species (8.74) |

**Table S9:** Top five bacteria identified at different taxonomic levels during the adult stages of *C. megacephala* collected on different feeding sources.

| Phylum (%) | Class (%) | Order (%) | Family (%) | Genus (%) | Species (%) |
|---|---|---|---|---|---|
| Proteobacteria (69.53)<br>Firmicutes (25.65)<br>Bacteroidota (4.37)<br>Actinobacteriota (0.42)<br>Fusobacteriota (0.002)<br>Other phyla (0.008) | Gammaproteobacteria (50.26)<br>Alphaproteobacteria (19.26)<br>Bacilli (16.45)<br>Clostridia (9.19)<br>Bacteroidia (4.37)<br>Other classes (0.43) | Cardiobacteriales (40.64)<br>Rickettsiales (18.92)<br>Lactobacillales (13.72)<br>Peptostreptococcales-Tissierellales (9.16)<br>Enterobacterales (6.08)<br>Other orders (11.46) | Wohlfahrtiimonadaceae (40.64)<br>Anaplasmataceae (18.92)<br>Family XI (8.09)<br>Vagococcaceae (5.80)<br>Lactobacillaceae (4.75)<br>Other families (21.78) | *Ignatzschineria* (4032)<br>*Wolbachia* (18.92)<br>*Vagococcus* (5.80)<br>*Weissella* (4.69)<br>*Providencia* (3.89)<br>Other genera (26.37) | *Ignatzschineria indica* (32.76)<br>*Wolbachia* spp. (18.92)<br>*Ignatzschineria ureiclastica* (6.64)<br>*Vagococcus* spp. (5.44)<br>*Weissella viridescens* (4.64)<br>Other species (31.57) |

**Table S10:** Top five bacteria identified at different taxonomic levels in the gut microbiome of *C. megacephala* across all temperature conditions.

| Development stages | Phylum (%) | Class (%) | Order (%) | Family (%) | Genus (%) | Species (%) |
|---|---|---|---|---|---|---|
| **Larvae** | Proteobacteria (70.72)<br>Firmicutes (29.17)<br>Actinobacteriota (0.05)<br>Bacteroidota (0.04)<br>Fusobacteriota (0.002)<br>Other phyla (0.0009) | Gammaproteobacteria (70.63)<br>Clostridia (19.15)<br>Bacilli (10.01)<br>Alphaproteobacteria (0.08)<br>Bacteroidia (0.04)<br>Other classes (0.06) | Cardiobacteriales (70.52)<br>Peptostreptococcales-Tissierellales (19.08)<br>Lactobacillales (8.39)<br>Bacillales (0.63)<br>Erysipelotrichales (0.55)<br>Other orders (0.80) | Wohlfahrtiimonadaceae (70.52)<br>Family_XI (17.51)<br>Vagococcaceae (7.47)<br>Peptostreptococcaceae (1.57)<br>Planococcaceae (0.63)<br>Other families (2.28) | *Ignatzschineria* 70.06)<br>*Gallicola* (7.60)<br>*Vagococcus* (7.47)<br>Unclassified *Family_XI* (6.04)<br>*Tissierella* (2.81)<br>Other genera (5.99) | *Ignatzschineria indica* (54.24)<br>*Ignatzschineria ureiclastica* (15.37)<br>*Gallicola* spp. (7.60)<br>*Vagococcus* spp. (7.40)<br>Unclassified *Family_XI* (6.04)<br>Other species (9.32) |
| **Pupae** | Proteobacteria (81.20)<br>Firmicutes (12.95)<br>Bacteroidota (5.50)<br>Actinobacteriota (0.31)<br>Fusobacteriota (0.005)<br>Other phyla (0.006) | Gammaproteobacteria (45.83)<br>Alphaproteobacteria (35.36)<br>Bacilli (7.71)<br>Bacteroidia (5.50)<br>Clostridia (5.23)<br>Other classes (0.33) | Cardiobacteriales (36.66)<br>Rickettsiales (35.07)<br>Enterobacterales (6.78)<br>Lactobacillales (5.51)<br>Peptostreptococcales-Tissierellales (5.22)<br>Other orders (10.73) | Wohlfahrtiimonadaceae (36.66)<br>Anaplasmataceae (35.07)<br>Morganellaceae (5.49)<br>Vagococcaceae (5.06)<br>Family_XI (3.95)<br>Other families (13.73) | *Ignatzschineria* (36.27)<br>*Wolbachia* (35.07)<br>*Vagococcus* (5.06)<br>*Providencia* (4.44)<br>Unclassified *Family_XI* (3.12)<br>Other genera (10.16) | *Wolbachia* spp. (35.07)<br>*Ignatzschineria indica* (32.50)<br>*Providencia* spp. (4.44)<br>*Vagococcus* spp. (4.34)<br>Unclassified *Family_XI* (3.12)<br>Other species (20.49) |
| **Adult** | Proteobacteria (50.74)<br>Firmicutes (38.88)<br>Bacteroidota (9.21)<br>Actinobacteriota (1.13)<br>Unclassified Bacteria (0.005)<br>Other phyla (0.01) | Bacilli (38.86)<br>Gammaproteobacteria (26.17)<br>Alphaproteobacteria (24.56)<br>Bacteroidia (9.21)<br>Actinobacteria (1.13)<br>Other classes (0.47) | Lactobacillales (33.69)<br>Rickettsiales (35.68)<br>Enterobacterales (14.08)<br>Flavobacteriales (9.10)<br>Pseudomonadales (8.89)<br>Other orders (10.47) | Anaplasmataceae (23.73)<br>Lactobacillaceae (18.60)<br>Enterococcaceae (10.31)<br>Morganellaceae (9.28)<br>Pseudomonadaceae (8.65)<br>Other families (29.40) | *Wolbachia* (23.73)<br>*Weissella* (18.39)<br>*Enterococcus* (10.31)<br>*Providencia* (8.87)<br>*Pseudomonas* (8.65)<br>Other genera (30.03) | *Wolbachia* spp. (24.08)<br>*Weissella viridescens* (18.47)<br>*Providencia* spp. (8.99)<br>*Enterococcus sulfureus* ATCC_49903 (7.87)<br>*Bacterium_28W232* (7.10)<br>Other species (34.91) |

**Table S11:** Top five bacteria identified at different taxonomic levels during the development stages of *C. megacephala* under overall temperature conditions.

| Temperature | Phylum (%) | Class (%) | Order (%) | Family (%) | Genus (%) | Species (%) |
|---|---|---|---|---|---|---|
| **LT** | Proteobacteria (90.21)<br>Firmicutes (9.76)<br>Bacteroidota (0.01)<br>Actinobacteriota (0.006) | Gammaproteobacteria (90.20)<br>Bacilli (7.95)<br>Clostridia (1.80)<br>Bacteroidia (0.01)<br>Actinobacteria (0.006)<br>Other classes (0.005) | Cardiobacteriales (90.19)<br>Lactobacillales (7.81)<br>Peptostreptococcales-Tissierellales (1.80)<br>Erysipelotrichales (0.12)<br>Bacteroidales (0.01)<br>Other orders (0.04) | Wohlfahrtiimonadaceae (90.19)<br>Vagococcaceae (7.61)<br>Family_XI (1.33)<br>Peptostreptococcaceae (0.46)<br>Streptococcaceae (0.17)<br>Other families (0.12) | *Ignatzschineria* (89.56)<br>*Vagococcus* (7.61)<br>*Wohlfahrtiimonas* (0.49)<br>*Gallicola* (0.47)<br>*Peptostreptococcus* (0.46)<br>Other genera (1.38) | *Ignatzschineria indica* (75.09)<br>*Ignatzschineria ureiclastica* (13.78)<br>*Vagococcus* spp. (7.53)<br>*Ignatzschineria* spp. (0.67)<br>*Wohlfahrtiimonas chitiniclastica* (0.49)<br>Other species (2.40) |
| **MT** | Proteobacteria (57.32)<br>Firmicutes (42.56)<br>Actinobacteriota (0.08)<br>Bacteroidota (0.01)<br>Acidobacteriota (0.0007)<br>Other phyla (0.001) | Gammaproteobacteria (57.31)<br>Clostridia (31.48)<br>Bacilli (11.07)<br>Actinobacteria (0.08)<br>Alphaproteobacteria (0.01)<br>Other classes (0.02) | Cardiobacteriales (57.17)<br>Peptostreptococcales-Tissierellales (31.46)<br>Lactobacillales (8.39)<br>Staphylococcales (1.51)<br>Erysipelotrichales (0.69)<br>Other orders (0.77) | Wohlfahrtiimonadaceae (57.17)<br>Family_XI (28.84)<br>Vagococcaceae (7.47)<br>Peptostreptococcaceae (2.60)<br>Staphylococcaceae (1.51)<br>Other families (2.39) | *Ignatzschineria* (57.16)<br>*Gallicola* (13.72)<br>Unclassified *Family_XI* (9.11)<br>*Vagococcus* (7.47)<br>*Tissierella* (5.43)<br>Other genera (7.10) | *Ignatzschineria indica* (39.60)<br>*Ignatzschineria ureiclastica* (17.53)<br>*Gallicola* spp. (13.72)<br>Unclassified *Family_XI* (9.11)<br>*Vagococcus* spp. (7.41)<br>Other species (12.61) |
| **HT** | Proteobacteria (5148)<br>Firmicutes (48.23)<br>Bacteroidota (0.14)<br>Actinobacteriota (0.13)<br>Deinococcota (0.0003)<br>Other phyla (0.001) | Gammaproteobacteria (51.17)<br>Clostridia (36.65)<br>Bacilli (11.58)<br>Alphaproteobacteria (0.30)<br>Bacteroidia (0.14)<br>Other classes (0.13) | Cardiobacteriales (50.93)<br>Peptostreptococcales-Tissierellales (36.55)<br>Lactobacillales (8.24)<br>Bacillales (2.04)<br>Erysipelotrichales (1.12)<br>Other orders (1.09) | Wohlfahrtiimonadaceae (50.93)<br>Family_XI (34.28)<br>Vagococcaceae (6.33)<br>Peptostreptococcaceae (2.26)<br>Planococcaceae (2.03)<br>Other families (4.13) | *Ignatzschineria* (50.92)<br>*Gallicola* (14.71)<br>unclassified *Family_XI* (12.10)<br>*Vagococcus* (6.33)<br>*Tissierella* (4.62)<br>Other genera (11.29) | *Ignatzschineria indica* (41.55)<br>*Gallicola* spp. (14.71)<br>unclassified *Family_XI* (12.10)<br>*Ignatzschineria ureiclastica* (9.34)<br>*Vagococcus* spp. (6.26)<br>Other species (16.01) |
| **VT** | Proteobacteria (83.86)<br>Firmicutes (16.11)<br>Bacteroidota (0.01)<br>Actinobacteriota (0.007)<br>Fusobacteriota (0.0007) | Gammaproteobacteria (83.85)<br>Bacilli (9.45)<br>Clostridia (6.65)<br>Bacteroidia (0.01)<br>Actinobacteria (0.007)<br>Other classes (0.004) | Cardiobacteriales (83.79)<br>Lactobacillales (9.12)<br>Peptostreptococcales-Tissierellales (6.54)<br>Erysipelotrichales (0.25)<br>Clostridiales (0.10)<br>Other orders (0.17) | Wohlfahrtiimonadaceae (83.79)<br>Vagococcaceae (8.48)<br>Family_XI (5.58)<br>Peptostreptococcaceae (0.95)<br>Streptococcaceae (0.45)<br>Other families (0.72) | *Ignatzschineria* (82.60)<br>*Vagococcus* (8.48)<br>Unclassified Family_XI (2.54)<br>*Wohlfahrtiimonas* (1.18)<br>*Gallicola* (1.84)<br>Other genera (3.68) | *Ignatzschineria indica* (60.71)<br>*Ignatzschineria ureiclastica* (20.83)<br>*Vagococcus* spp. (8.40)<br>unclassified *Family_XI* (2.54)<br>*Gallicola* spp. (1.50)<br>Other species (5.99) |

**Table S12:** Top five bacteria identified at different taxonomic levels during the third larval stages of *C. megacephala,* reared under different constant and variable temperatures.

| Temperature | Phylum (%) | Class (%) | Order (%) | Family (%) | Genus (%) | Species (%) |
|---|---|---|---|---|---|---|
| **LT** | Proteobacteria (91.26)<br>Firmicutes (6.55)<br>Bacteroidota (1.65)<br>Actinobacteriota (0.52)<br>Chloroflexi (0.0003) | Gammaproteobacteria (49.76)<br>Alphaproteobacteria (41.50)<br>Bacilli (5.27)<br>Bacteroidia (1.65)<br>Clostridia (1.27)<br>Other classes (0.52) | Rickettsiales (41.20)<br>Cardiobacteriales (38.08)<br>Enterobacterales (9.70)<br>Lactobacillales (4.98)<br>Pseudomonadales (1.48)<br>Other orders (4.53) | Anaplasmataceae (41.20)<br>Wohlfahrtiimonadaceae (38.08)<br>Morganellaceae (7.32)<br>Vagococcaceae (4.96)<br>Hafniaceae (1.70)<br>Other families (6.72) | *Wolbachia* (41.20)<br>*Ignatzschineria* (37.98)<br>*Providencia* (7.11)<br>*Vagococcus* (4.96)<br>*Hafnia-Obesumbacterium* (1.70)<br>Other genera (7.03) | *Wolbachia* spp. (41.20)<br>*Ignatzschineria indica* (37.49)<br>*Providencia* spp. (7.11)<br>*Vagococcus* spp. (4.80)<br>*Hafnia alvei* (1.70)<br>Other species (7.68) |
| **MT** | Proteobacteria (76.98)<br>Firmicutes (21.28)<br>Bacteroidota (1.53)<br>Actinobacteriota (0.17)<br>Fusobacteriota (0.01)<br>Other phyla (0.005) | Alphaproteobacteria (41.87)<br>Gammaproteobacteria (35.10)<br>Bacilli (12.96)<br>Clostridia (8.30)<br>Bacteroidia (1.53)<br>(1.13)<br>Other classes (0.20) | Rickettsiales (41.76)<br>Cardiobacteriales (29.20)<br>Lactobacillales (10.33)<br>Peptostreptococcales-Tissierellales (8.30)<br>Enterobacterales (5.18)<br>Other orders (5.20) | Anaplasmataceae (41.76)<br>Wohlfahrtiimonadaceae (29.20)<br>Vagococcaceae (10.11)<br>Morganellaceae (5.05)<br>Peptostreptococcaceae (4.75)<br>Other families (9.11) | *Wolbachia* (41.76)<br>*Ignatzschineria* (28.76)<br>*Vagococcus* (10.11)<br>*Providencia* (4.22)<br>*Paraclostridium* (3.50)<br>Other genera (11.62) | *Wolbachia* spp. (41.76)<br>*Ignatzschineria indica* (23.83)<br>*Vagococcus* spp. (8.54)<br>*Providencia* spp. (4.22)<br>*Paraclostridium bifermentans* (3.50)<br>Other species (18.11) |
| **HT** | Proteobacteria (79.34)<br>Firmicutes (12.30)<br>Bacteroidota (8.18)<br>Actinobacteriota (0.15)<br>Chloroflexi (0.004)<br>Other phyla (0.008) | Gammaproteobacteria (49.68)<br>Bacilli (26.31)<br>Clostridia (15.95)<br>Actinobacteria (5.30)<br>Bacteroidia (2.26)<br>Other classes (0.46) | Cardiobacteriales (46.32)<br>Rickettsiales (18.10)<br>Enterobacterales (9.30)<br>Flavobacteriales (7.47)<br>Lactobacillales (4.52)<br>Other orders (14.25) | Wohlfahrtiimonadaceae (46.32)<br>Anaplasmataceae (18.10)<br>Weeksellaceae (7.39)<br>Morganellaceae (6.78)<br>Planococcaceae (3.27)<br>Other families (18.10) | *Ignatzschineria* (46.32)<br>*Wolbachia* (18.10)<br>*Chryseobacterium* (7.37)<br>*Providencia* (5.21)<br>*Vagococcus* (2.91)<br>Other genera (20.06) | *Ignatzschineria indica* (45.23)<br>*Wolbachia* spp. (18.10)<br>*Chryseobacterium indologenes*. (7.37)<br>*Providencia* spp. (5.21)<br>Unclassified *Family_XI* (2.16)<br>Other species (21.91) |
| **VT** | Proteobacteria (77.02)<br>Firmicutes (11.62)<br>Bacteroidota (10.96)<br>Actinobacteriota (0.37)<br>Fusobacteriota (0.006)<br>Other phyla (0.008) | Gammaproteobacteria (39.52)<br>Alphaproteobacteria (37.49)<br>Bacteroidia (10.96)<br>Clostridia (8.15)<br>Bacilli (3.46)<br>Other classes (0.39) | Rickettsiales (37.33)<br>Cardiobacteriales (34.09)<br>Peptostreptococcales-Tissierellales (8.12)<br>Bacteroidales (6.32)<br>Flavobacteriales (4.62)<br>Other orders (9.48) | Anaplasmataceae (37.33)<br>Wohlfahrtiimonadaceae (34.09)<br>Family_XI (8.05)<br>Bacteroidaceae (6.31)<br>Flavobacteriaceae (4.54)<br>Other families (9.64) | *Wolbachia* (37.33)<br>*Ignatzschineria* (33.16)<br>Unclassified *Family_XI* (6.79)<br>*Bacteroides* (6.31)<br>*Myroides* (4.54)<br>Other genera (11.83) | *Wolbachia* spp. (37.33)<br>*Ignatzschineria indica* (24.89)<br>Unclassified *Family_XI* (6.79)<br>*Bacteroides* spp. (6.06)<br>*Bacterium 28W232* (4.50)<br>Other species (20.40) |

**Table S13:** Top five bacteria identified at different taxonomic levels during the pupal stages of *C. megacephala,* raised at different constant and variable temperatures.

| Temperature | Phylum (%) | Class (%) | Order (%) | Family (%) | Genus (%) | Species (%) |
|---|---|---|---|---|---|---|
| **LT** | Proteobacteria (68.14)<br>Firmicutes (26.94)<br>Actinobacteriota (2.75)<br>Bacteroidota (2.12)<br>Unclassified Bacteria (0.01)<br>Other phyla (0.01) | Gammaproteobacteria (40.54)<br>Alphaproteobacteria (27.59)<br>Bacilli (26.92)<br>Actinobacteria (2.75)<br>Bacteroidia (2.12)<br>Other classes (0.05) | Enterobacterales (34.65)<br>Lactobacillales (26.85)<br>Rickettsiales (25.79)<br>Corynebacteriales (2.69)<br>Cardiobacteriales (2.60)<br>Other orders (7.40) | Morganellaceae (28.36)<br>Anaplasmataceae (25.79)<br>Lactobacillaceae (23.36)<br>Enterococcaceae (3.43)<br>Nocardiaceae (2.69)<br>Other families (16.34) | *Providencia* (28.22)<br>*Wolbachia* (25.79)<br>*Weissella* (23.34)<br>*Enterococcus* (3.43)<br>*Rhodococcus* (2.67)<br>Other genera (16.52) | *Providencia* spp. (28.21)<br>*Wolbachia* spp. (25.79)<br>*Weissella viridescens* (23.27)<br>*Enterococcus sulfureus* ATCC 49903 (3.42)<br>*Rhodococcus erythropolis* (2.67)<br>Other species (16.60) |
| **MT** | Proteobacteria (58.02)<br>Firmicutes (40.71)<br>Bacteroidota (0.88)<br>Actinobacteriota (0.33)<br>Deinococcota (0.006)<br>Other phyla (0.03) | Bacilli (40.67)<br>Alphaproteobacteria (33.79)<br>Gammaproteobacteria (24.23)<br>Bacteroidia (0.88)<br>Actinobacteria (0.33)<br>Other classes (0.08) | Rickettsiales (33.20)<br>Lactobacillales (26.95)<br>Pseudomonadales (20.53)<br>Staphylococcales (13.63)<br>Burkholderiales (1.74)<br>Other orders (3.91) | Anaplasmataceae (33.20)<br>Peudomonadaceae (20.23)<br>Enterococcaceae (15.27)<br>Staphylococcaceae (13.63)<br>Vagococcaceae (7.41)<br>Other families (10.23) | *Wolbachia* (33.20)<br>*Pseudomonas* (20.23)<br>*Enterococcus* (15.27)<br>*Staphylococcus* (13.59)<br>*Vagococcus* (7.41)<br>Other genera (10.27) | *Wolbachia* spp. (33.20)<br>*Gammaproteobacteria bacterium*_ESL0073 (19.63)<br>*Weissella viridescens* (15.16)<br>*Enterococcus sulfureus* ATCC 49903 (15.18)<br>*Staphylococcus nepalensis*. (11.94)<br>*Vagococcus* spp. (7.10)<br>Other species (12.91) |
| **HT** | Proteobacteria (52.75)<br>Firmicutes (41.25)<br>Bacteroidota (5.43)<br>Actinobacteriota (0.55)<br>Cyanobacteria (0.003)<br>Other phyla (0.004) | Bacilli (41.23)<br>Gammaproteobacteria (35.12)<br>Alphaproteobacteria (17.62)<br>Bacteroidia (5.43)<br>Actinobacteria (0.55)<br>Other classes (0.03) | Lactobacillales (35.17)<br>Enterobacterales (20.38)<br>Rickettsiales (16.84)<br>Pseudomonadales (10.26)<br>Staphylococcales (6.03)<br>Other orders (11.30) | Anaplasmataceae (16.84)<br>Lactobacillaceae (14.29)<br>Enterococcaceae (11.98)<br>Enterobacteriaceae (11.77)<br>Pseudomonadaceae (10.05)<br>Other families (35.05) | *Wolbachia* (16.84)<br>*Weissella* (14.21)<br>*Enterococcus* (11.98)<br>*Enterobacter* (11.56)<br>*Pseudomonas* (10.05)<br>Other genera (35.34) | *Wolbachia* spp. (16.84)<br>*Weissella viridescens* (13.96)<br>*Enterococcus faecalis* (9.65)<br>*Klebsiella quasipneumoniae* (9.15)<br>*Vagococcus* spp. (8.25)<br>Other species (42.13) |
| **VT** | Firmicutes (46.64)<br>Bacteroidota (28.40)<br>Proteobacteria (24.04)<br>Actinobacteriota (0.89)<br>Unclassified Bacteria (0.002)<br>Other phyla (0.0005) | Bacilli (46.63)<br>Bacteroidia (28.40)<br>Alphaproteobacteria (19.24)<br>Gammaproteobacteria (4.80)<br>Actinobacteria (0.89)<br>Other classes (0.01) | Lactobacillales (45.81)<br>Flavobacteriales (28.30)<br>Rickettsiales (19.09)<br>Pseudomonadales (3.31)<br>Staphylococcales (0.81)<br>Other orders (2.65) | Lactobacillaceae (33.55)<br>Flavobacteriaceae (28.05)<br>Anaplasmataceae (19.09)<br>Enterococcaceae (10.54)<br>Pseudomonadaceae (3.27)<br>Other families (5.47) | *Weissella* (33.38)<br>*Myroides* (28.03)<br>*Wolbachia* (19.09)<br>*Enterococcus* (10.54)<br>*Pseudomonas* (3.27)<br>Other genera (5.66) | *Weissella viridescens* (32.99)<br>*Bacterium 28W232* (28.02)<br>*Wolbachia* spp. (19.09)<br>*Enterococcus sulfureus* ATCC 49903 (10.09)<br>*Gammaproteobacteria bacterium* ESL0073 (1.70)<br>Other species (8.07) |

**Table S14:**Top five bacteria found at different taxonomic levels during the adult stages of *C. megacephala,* reared under different constant and variable temperatures.

| Temperature | Phylum (%) | Class (%) | Order (%) | Family (%) | Genus (%) | Species (%) |
|---|---|---|---|---|---|---|
| **LT** | Proteobacteria (85.09)<br>Firmicutes (12.85)<br>Bacteroidota (1.15)<br>Actinobacteriota (0.88)<br>Unclassified Bacteria (0.004)<br>Other phyla (0.04) | Gammaproteobacteria (62.62)<br>Alphaproteobacteria (22.46)<br>Bacilli (11.69)<br>Clostridia (1.15)<br>Bacteroidia (1.15)<br>Other classes (0.89) | Cardiobacteriales (48.75)<br>Rickettsiales (21.90)<br>Enterobacterales (12.30)<br>Lactobacillales (11.51)<br>Peptostreptococcales-Tissierellales (1.15)<br>Other orders (7.40) | Wohlfahrtiimonadaceae (48.75)<br>Anaplasmataceae (21.90)<br>Morganellaceae (9.83)<br>Lactobacillaceae (5.84)<br>Vagococcaceae (4.72)<br>Other families (9.83) | *Ignatzschineria* (48.47)<br>*Wolbachia* (21.90)<br>*Providencia* (9.72)<br>*Weissella* (5.83)<br>*Vagococcus* (4.72)<br>Other genera (9.33) | *Ignatzschineria indica* (42.32)<br>*Wolbachia* spp. (21.90)<br>*Providencia* spp. (9.72)<br>*Weissella viridescens* (5.82)<br>*Ignatzschineria ureiclastica* (5.75)<br>Other species (14.47) |
| **MT** | Proteobacteria (64.87)<br>Firmicutes (34.12)<br>Bacteroidota (0.80)<br>Actinobacteriota (0.18)<br>Fusobacteriota (0.006)<br>Other phyla (0.01) | Gammaproteobacteria (40.71)<br>Alphaproteobacteria (24.15)<br>Bacilli (19.18)<br>Clostridia (14.93)<br>Bacteroidia (0.80)<br>Other classes (0.20) | Cardiobacteriales (32.73)<br>Rickettsiales (23.96)<br>Peptostreptococcales-Tissierellales (14.91)<br>Lactobacillales (13.76)<br>Pseudomonadales (5.34)<br>Other orders (9.27) | Wohlfahrtiimonadaceae (32.73)<br>Anaplasmataceae (23.96)<br>Family_XI (12.15)<br>Vagococcaceae (8.44)<br>Pseudomonadaceae (5.16)<br>Other families (17.53) | *Ignatzschineria* (32.56)<br>*Wolbachia* (23.96)<br>*Vagococcus* (8.44)<br>*Gallicola* (5.32)<br>*Pseudomonas* (5.14)<br>Other genera (24.55) | *Ignatzschineria indica* (24.07)<br>*Wolbachia* spp. (23.96)<br>*Vagococcus* spp. (7.76)<br>*Ignatzschineria ureiclastica* (7.44)<br>*Gallicola* spp. (5.32)<br>Other species (31.42) |
| **HT** | Proteobacteria (61.50)<br>Firmicutes (33.91)<br>Bacteroidota (4.31)<br>Actinobacteriota (0.25)<br>Cyanobacteria (0.001)<br>Other phyla (0.005) | Gammaproteobacteria (50.27)<br>Bacilli (18.53)<br>Clostridia (15.38)<br>Alphaproteobacteria (11.23)<br>Bacteroidia (4.31)<br>Other classes (0.26) | Cardiobacteriales (36.55)<br>Peptostreptococcales-Tissierellales (15.33)<br>Lactobacillales (13.97)<br>Rickettsiales (10.69)<br>Enterobacterales (8.60)<br>Other orders (14.83) | Wohlfahrtiimonadaceae (36.55)<br>Family_X (14.43)<br>Anaplasmataceae (10.69)<br>Vagococcaceae (5.76)<br>Morganellaceae (4.62)<br>Other families (27.92) | *Ignatzschineria* (36.54)<br>*Wolbachia* (10.69)<br>*Gallicola* (5.80)<br>*Vagococcus* (5.76)<br>Unclassified *Family_XI* (5.49)<br>Other genera (36.69) | *Ignatzschineria indica* (32.44)<br>*Wolbachia* spp. (10.69)<br>*Gallicola* spp. (5.80)<br>Unclassified *Family_XI* (5.49)<br>*Vagococcus* spp. (5.25)<br>Other species (40.30) |
| **VT** | Proteobacteria (66.34)<br>Firmicutes (22.06)<br>Bacteroidota (11.21)<br>Actinobacteriota (0.36)<br>Fusobacteriota (0.002)<br>Other phyla (0.04) | Gammaproteobacteria (47.46)<br>Alphaproteobacteria (18.87)<br>Bacilli (16.50)<br>Bacteroidia (11.21)<br>Clostridia (5.55)<br>Other classes (0.37) | Cardiobacteriales (44.34)<br>Rickettsiales (18.77)<br>Lactobacillales (15.66)<br>Flavobacteriales (8.80)<br>Peptostreptococcales-Tissierellales (5.50)<br>Other orders (6.90) | Wohlfahrtiimonadaceae (44.34)<br>Anaplasmataceae (18.77)<br>Flavobacteriaceae (8.71)<br>Lactobacillaceae (8.40)<br>Family XI (5.11)<br>Other families (14.64) | *Ignatzschineria* (43.54)<br>*Wolbachia* (18.77)<br>*Myroides* (8.71)<br>*Weissella* (8.35)<br>*Vagococcus* (4.27)<br>Other genera (16.33) | *Ignatzschineria indica* (32.21)<br>*Wolbachia* spp. (18.77)<br>*Ignatzschineria ureiclastica* (9.35)<br>*Bacterium 28W232* (8.69)<br>*Weissella viridescens* (8.25)<br>Other species (22.70) |

**Table S15:** Global view of top five bacteria identified at different taxonomic levels in response to different temperature treatments on the gut of *C. megacephala.*

| Temperature | | LT | | | MT | | | HT | | | VT | | |
|---|---|---|---|---|---|---|---|---|---|---|---|---|---|
| Development stages | | | | | | | | | | | | | |
| Phenotype | Taxon on genus level | Larvae | Pupae | Adult | Larvae | Pupae | Adult | Larvae | Pupae | Adult | Larvae | Pupae | Adult |
| Aerobic | *Wolbachia* | 0.00056 | 0.94436 | 0.68442 | 0.00051 | 0.86430 | 0.87747 | 0.00016 | 0.75698 | 0.58888 | 0.00000 | 0.86479 | 0.70501 |
| | *Ignatzschineria* | 0.92236 | 0.00324 | 0.03035 | 0.78568 | 0.05043 | 0.00258 | 0.65060 | 0.01978 | 0.00250 | 0.88373 | 0.09071 | 0.00139 |
| | *Weissella* | 0.00005 | 0.00000 | 0.13405 | 0.00765 | 0.00000 | 0.01375 | 0.00005 | 0.00000 | 0.09768 | 0.00000 | 0.00009 | 0.24362 |
| | *Wohlfahrtiimonas* | 0.03333 | 0.00099 | 0.00010 | 0.00074 | 0.00434 | 0.00000 | 0.00064 | 0.00002 | 0.00004 | 0.05018 | 0.01059 | 0.00002 |
| | *Pseudomonas* | 0.00000 | 0.00461 | 0.00260 | 0.00002 | 0.00047 | 0.00230 | 0.00015 | 0.01403 | 0.05223 | 0.00001 | 0.00308 | 0.00951 |
| Gram Negative | *Wolbachia* | 0.00056 | 0.94436 | 0.68442 | 0.00051 | 0.86430 | 0.87747 | 0.00016 | 0.75698 | 0.58888 | 0.00000 | 0.86479 | 0.70501 |
| | *Ignatzschineria* | 0.92236 | 0.00324 | 0.03035 | 0.78568 | 0.05043 | 0.00258 | 0.65060 | 0.01978 | 0.00250 | 0.88373 | 0.09071 | 0.00139 |
| | *Providencia* | 4.37E-05 | 0.02324 | 0.05789 | 0.00026 | 0.01248 | 3.89E-05 | 0.00226 | 0.03110 | 0.03546 | 5.09E-05 | 0.00435 | 0.00075 |
| | *Chryseobacterium* | 0.00000 | 0.00339 | 0.00232 | 5.99E-05 | 0.00084 | 0.00372 | 5.31E-05 | 0.07710 | 0.04573 | 0.00000 | 8.62E-05 | 0.00023 |
| | *Wohlfahrtiimonas* | 0.03333 | 0.00099 | 9.90E-05 | 0.00074 | 0.00434 | 0.00000 | 0.00064 | 1.80E-05 | 4.01E-05 | 0.05018 | 0.01059 | 2.11E-05 |
| Forms Biofilms | *Wolbachia* | 0.00056 | 0.94436 | 0.68442 | 0.00051 | 0.86430 | 0.87747 | 0.00016 | 0.75698 | 0.58888 | 0.00000 | 0.86479 | 0.70501 |
| | *Ignatzschineria* | 0.92236 | 0.00324 | 0.03035 | 0.78568 | 0.05043 | 0.00258 | 0.65060 | 0.01978 | 0.00250 | 0.88373 | 0.09071 | 0.00139 |
| | *Providencia* | 0.00004 | 0.02324 | 0.05789 | 0.00026 | 0.01248 | 0.00004 | 0.00226 | 0.03110 | 0.03546 | 0.00005 | 0.00435 | 0.00075 |
| | *Wohlfahrtiimonas* | 0.03333 | 0.00099 | 0.00010 | 0.00074 | 0.00434 | 0.00000 | 0.00064 | 0.00002 | 0.00004 | 0.05018 | 0.01059 | 0.00002 |
| | *Pseudomonas* | 0.00000 | 0.00461 | 0.00260 | 0.00002 | 0.00047 | 0.00230 | 0.00015 | 0.01403 | 0.05223 | 0.00001 | 0.00308 | 0.00951 |
| Potentially Pathogenic | *Ignatzschineria* | 0.92236 | 0.00324 | 0.03035 | 0.78568 | 0.05043 | 0.00258 | 0.65060 | 0.01978 | 0.00250 | 0.88373 | 0.09071 | 0.00139 |
| | *Providencia* | 0.00004 | 0.02324 | 0.05789 | 0.00026 | 0.01248 | 0.00004 | 0.00226 | 0.03110 | 0.03546 | 0.00005 | 0.00435 | 0.00075 |
| | *Wohlfahrtiimonas* | 0.03333 | 0.00099 | 0.00010 | 0.00074 | 0.00434 | 0.00000 | 0.00064 | 0.00002 | 0.00004 | 0.05018 | 0.01059 | 0.00002 |
| | *Pseudomonas* | 0.00000 | 0.00461 | 0.00260 | 0.00002 | 0.00047 | 0.00230 | 0.00015 | 0.01403 | 0.05223 | 0.00001 | 0.00308 | 0.00951 |
| | *Enterobacter* | 0.00000 | 0.00221 | 0.00132 | 0.00000 | 0.00026 | 0.00074 | 0.00011 | 0.00896 | 0.03934 | 0.00000 | 0.00045 | 0.00102 |
| Stress Tolerant | *Ignatzschineria* | 0.92236 | 0.00324 | 0.03035 | 0.78568 | 0.05043 | 0.00258 | 0.65060 | 0.01978 | 0.00250 | 0.88373 | 0.09071 | 0.00139 |
| | *Providencia* | 0.00004 | 0.02324 | 0.05789 | 0.00026 | 0.01248 | 0.00004 | 0.00226 | 0.03110 | 0.03546 | 0.00005 | 0.00435 | 0.00075 |
| | *Wohlfahrtiimonas* | 0.03333 | 0.00099 | 0.00010 | 0.00074 | 0.00434 | 0.00000 | 0.00064 | 0.00002 | 0.00004 | 0.05018 | 0.01059 | 0.00002 |
| | *Pseudomonas* | 0.00000 | 0.00461 | 0.00260 | 0.00002 | 0.00047 | 0.00230 | 0.00015 | 0.01403 | 0.05223 | 0.00001 | 0.00308 | 0.00951 |
| | *Enterobacter* | 0.00000 | 0.00221 | 0.00132 | 0.00000 | 0.00026 | 0.00074 | 0.00011 | 0.00896 | 0.03934 | 0.00000 | 0.00045 | 0.00102 |

| | | | | | | | | | | | | | |
|---|---|---|---|---|---|---|---|---|---|---|---|---|---|
| Contains mobile elements | *Weissella* | 0.00005 | 0.00000 | 0.13405 | 0.00765 | 0.00000 | 0.01375 | 0.00005 | 0.00000 | 0.09768 | 0.00000 | 0.00009 | 0.24362 |
| | *Peptostreptococcus* | 0.03080 | 0.00074 | 0.00000 | 0.11331 | 0.01282 | 0.00006 | 0.11479 | 0.00052 | 0.00000 | 0.02887 | 0.00060 | 0.00002 |
| | *Providencia* | 0.00004 | 0.02324 | 0.05789 | 0.00026 | 0.01248 | 0.00004 | 0.00226 | 0.03110 | 0.03546 | 0.00005 | 0.00435 | 0.00075 |
| | *Staphylococcus* | 0.00000 | 0.00008 | 0.00011 | 0.01519 | 0.00034 | 0.06348 | 0.00067 | 0.00175 | 0.02030 | 0.00000 | 0.00013 | 0.00297 |
| | *Pseudomonas* | 0.00000 | 0.00450 | 0.00250 | 0.00002 | 0.00047 | 0.00211 | 0.00015 | 0.01342 | 0.05194 | 0.00001 | 0.00300 | 0.00926 |
| Gram Positive | *Weissella* | 0.00005 | 0.00000 | 0.13405 | 0.00765 | 0.00000 | 0.01375 | 0.00005 | 0.00000 | 0.09768 | 0.00000 | 0.00009 | 0.24362 |
| | *Peptostreptococcus* | 0.03080 | 0.00074 | 0.00000 | 0.11338 | 0.01284 | 0.00006 | 0.11580 | 0.00052 | 0.00000 | 0.02921 | 0.00060 | 0.00002 |
| | *Tissierella* | 0.00332 | 0.00006 | 0.00000 | 0.05483 | 0.00031 | 0.00000 | 0.07498 | 0.00084 | 0.00001 | 0.00790 | 0.00051 | 0.00000 |
| | *Staphylococcus* | 0.00000 | 0.00008 | 0.00011 | 0.01519 | 0.00034 | 0.06348 | 0.00067 | 0.00175 | 0.02030 | 0.00000 | 0.00013 | 0.00297 |
| | *Paraclostridium* | 0.00018 | 0.00007 | 0.00000 | 0.00226 | 0.03627 | 0.00003 | 0.03035 | 0.00006 | 0.00000 | 0.00791 | 0.00018 | 0.00003 |
| Anaerobic | *Peptostreptococcus* | 0.03080 | 0.00074 | 0.00000 | 0.11338 | 0.01284 | 0.00006 | 0.11580 | 0.00052 | 0.00000 | 0.02921 | 0.00060 | 0.00002 |
| | *Tissierella* | 0.00332 | 0.00006 | 0.00000 | 0.05483 | 0.00031 | 0.00000 | 0.07498 | 0.00084 | 0.00001 | 0.00790 | 0.00051 | 0.00000 |
| | *Paraclostridium* | 0.00018 | 0.00007 | 0.00000 | 0.00226 | 0.03627 | 0.00003 | 0.03035 | 0.00006 | 0.00000 | 0.00791 | 0.00018 | 0.00003 |
| | *Bacteroides* | 0.00000 | 0.00000 | 0.00000 | 0.00005 | 0.00010 | 0.00000 | 0.00366 | 0.00533 | 0.00000 | 0.00003 | 0.00184 | 0.00000 |
| | *Peptoniphilus* | 0.00139 | 0.00030 | 0.00000 | 0.00046 | 0.00050 | 0.00000 | 0.00005 | 0.00000 | 0.00000 | 0.00457 | 0.00023 | 0.00000 |
| Facultatively Anaerobic | *Providencia* | 0.00004 | 0.02324 | 0.05789 | 0.00026 | 0.01248 | 0.00004 | 0.00226 | 0.03110 | 0.03546 | 0.00005 | 0.00435 | 0.00075 |
| | *Staphylococcus* | 0.00000 | 0.00008 | 0.00011 | 0.01519 | 0.00034 | 0.06348 | 0.00067 | 0.00175 | 0.02030 | 0.00000 | 0.00013 | 0.00297 |
| | *Enterobacter* | 0.00000 | 0.00221 | 0.00132 | 0.00000 | 0.00026 | 0.00074 | 0.00011 | 0.00896 | 0.03934 | 0.00000 | 0.00045 | 0.00102 |
| | *Streptococcus* | 0.00393 | 0.00001 | 0.00000 | 0.00189 | 0.00000 | 0.00036 | 0.03991 | 0.00001 | 0.00000 | 0.00627 | 0.00002 | 0.00001 |
| | *Savagea* | 0.00004 | 0.00004 | 0.00000 | 0.00367 | 0.00099 | 0.00006 | 0.02817 | 0.00457 | 0.00000 | 0.00020 | 0.00106 | 0.00000 |

**Table S16:** Phenotype of top five bacterial genera at different development stages of *C. megacephala* raised at different constant and variable temperatures.
