# Supplementary figures and images for: Effects of Feeding Sources and Different Temperature Changes on the Gut Microbiome Structure of Chrysomya megacephala (Diptera: Calliphoridae)
Source: Insects. 2025 Mar 8;16(3):283. doi: 10.3390/insects16030283 (PMC11943086; doi:10.3390/insects16030283)

## Shannon curves

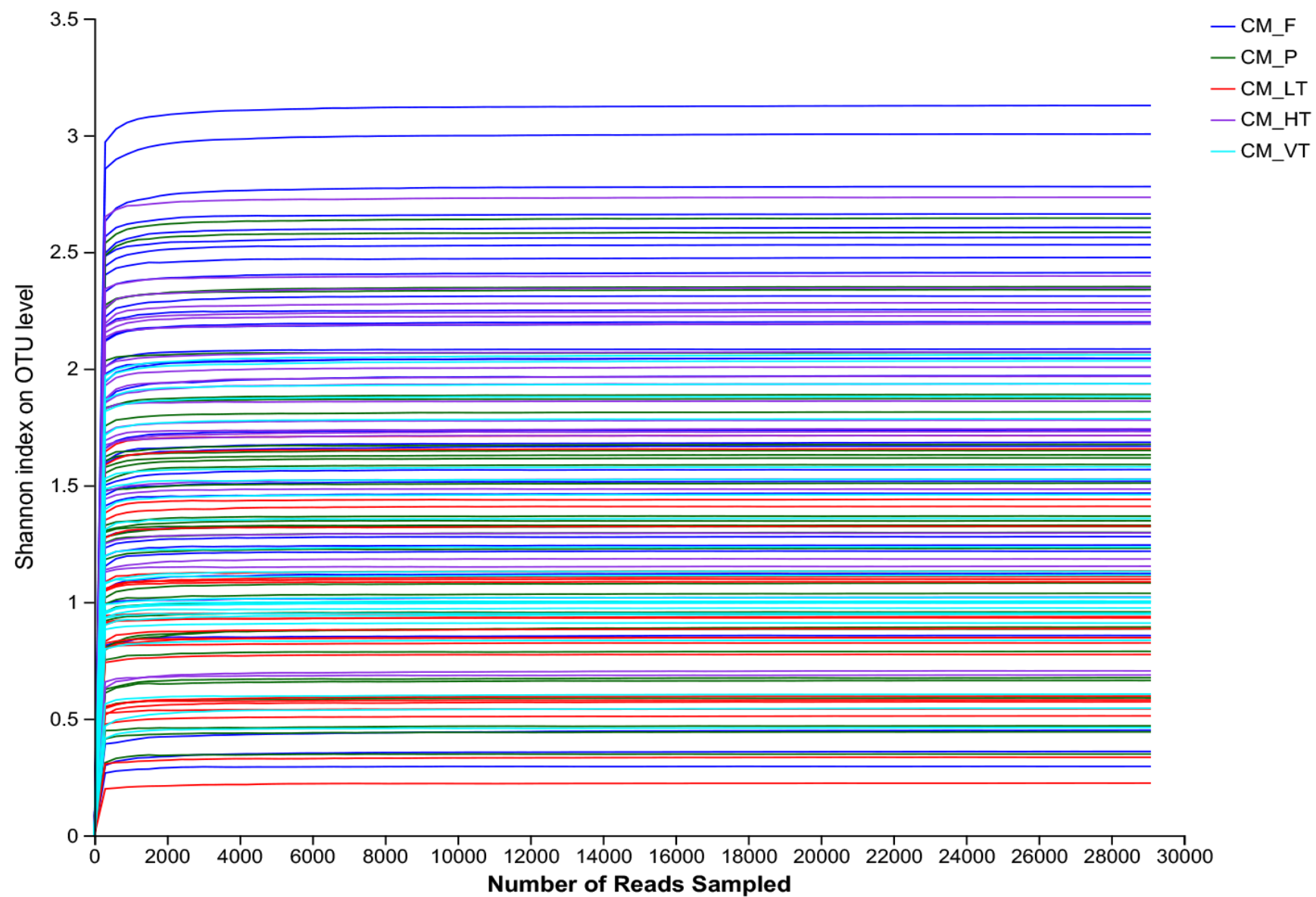

Supplement: Supplementary file 1 [file insects-16-00283-s001.zip › insects-3434865-supplementary/supplementary files/Figure S1.pdf]

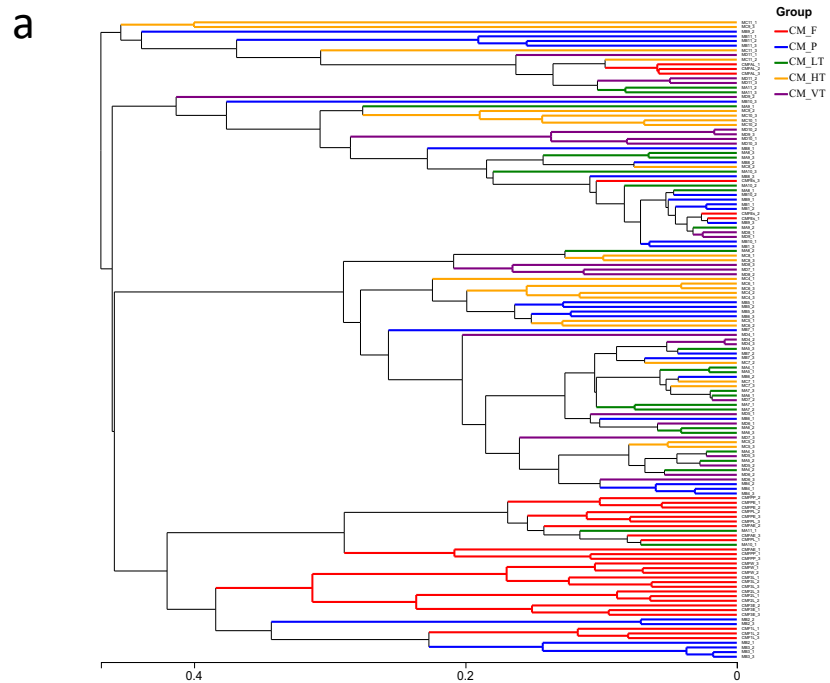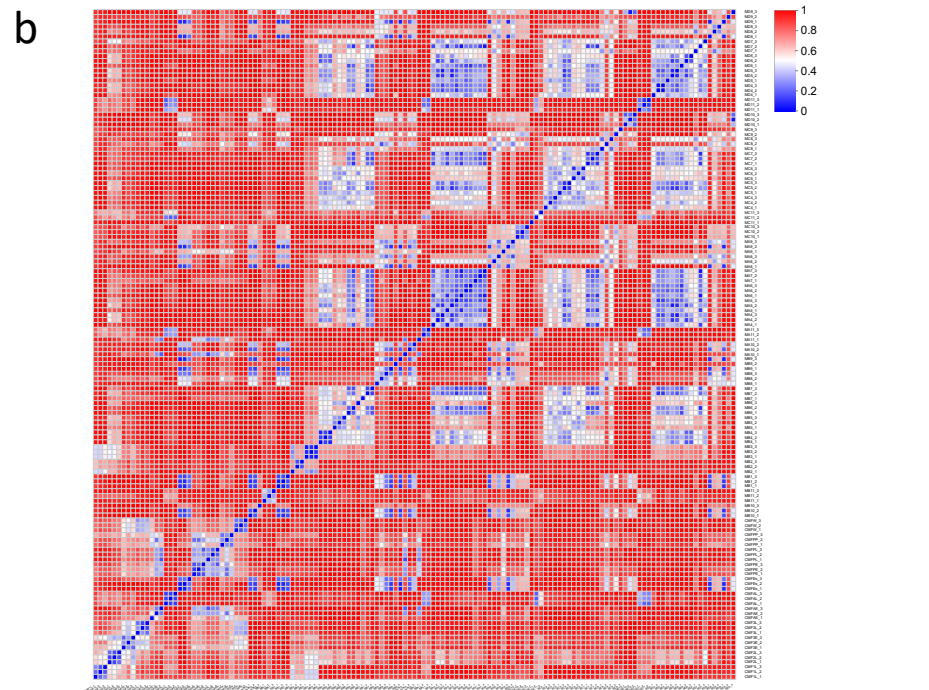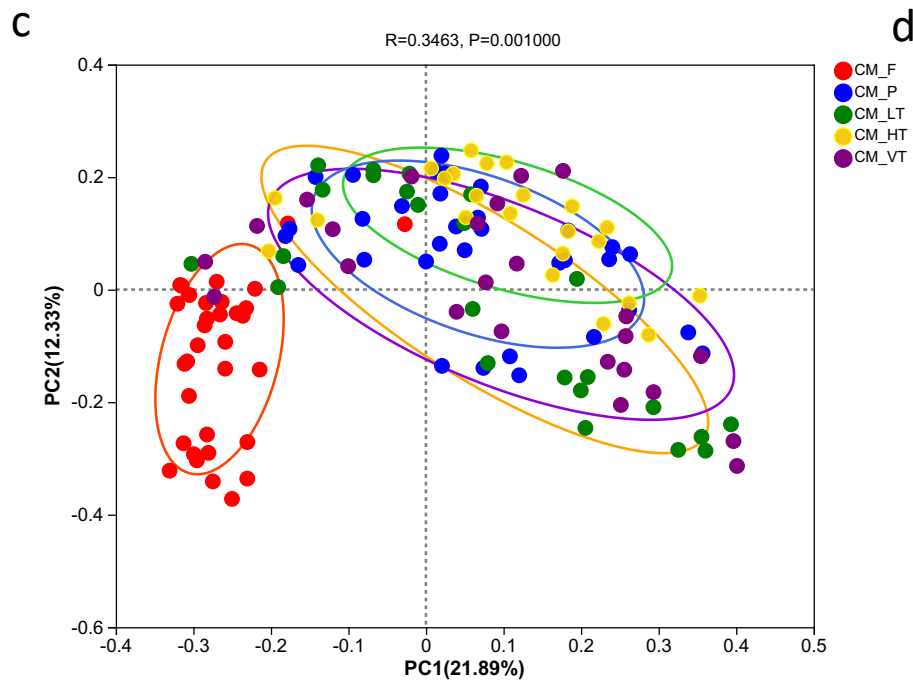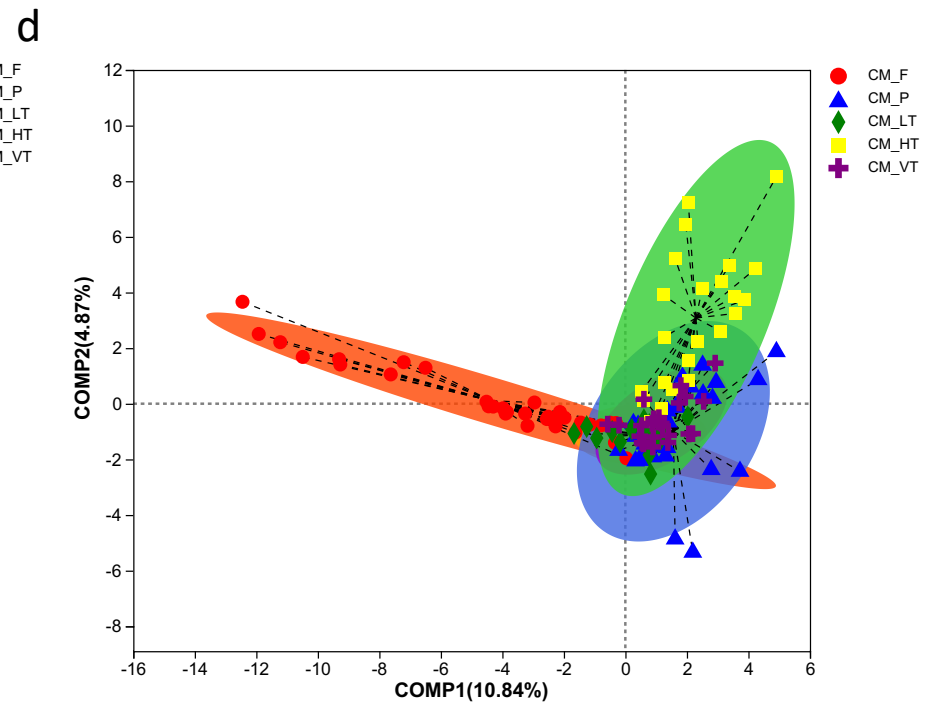

Supplement: Supplementary file 1 [file insects-16-00283-s001.zip › insects-3434865-supplementary/supplementary files/Figure S2.pdf]

# Phylogenetic tree on Genus level

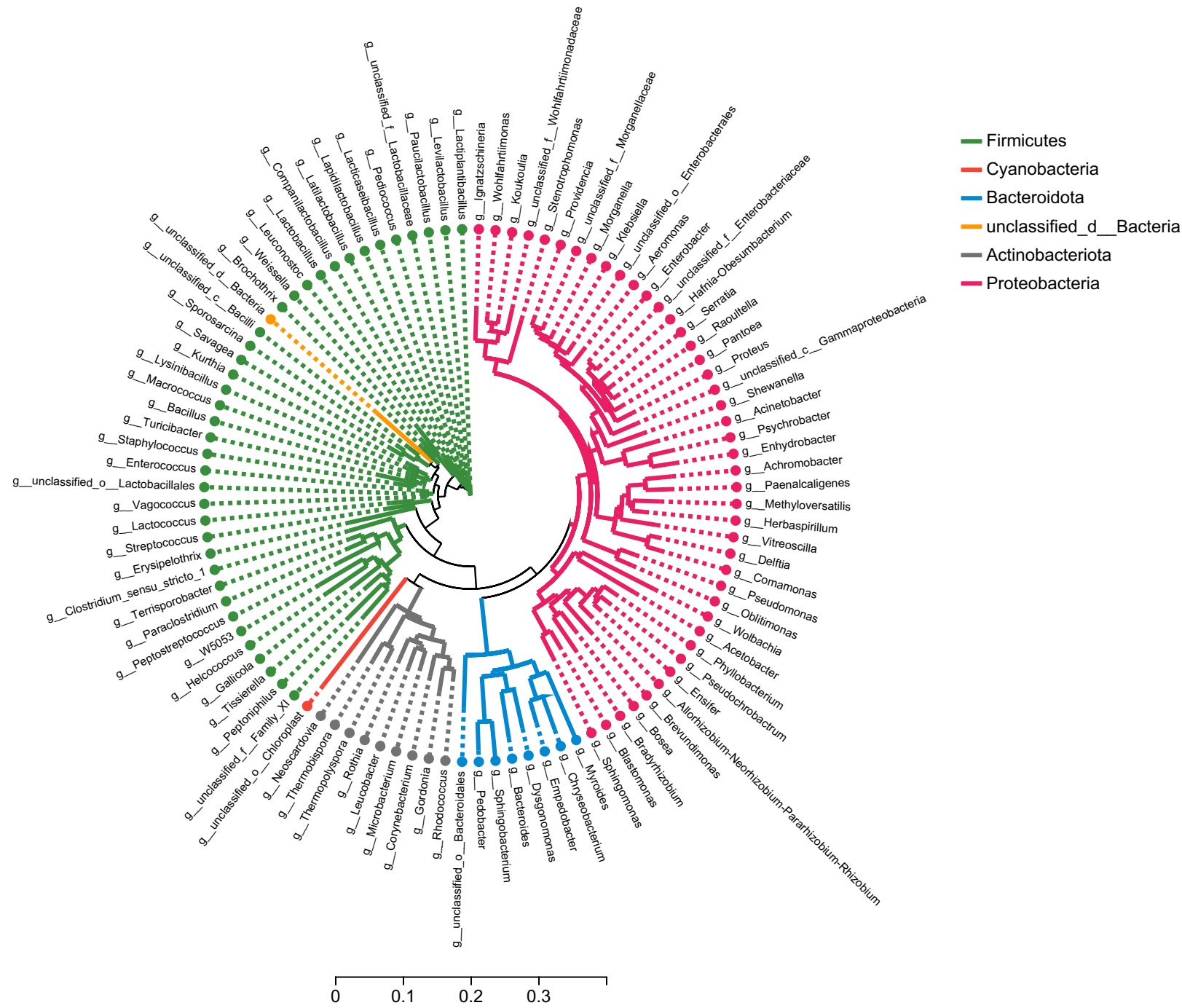

Supplement: Supplementary file 1 [file insects-16-00283-s001.zip › insects-3434865-supplementary/supplementary files/Figure S5.pdf]
